# Supplementary material for: Autophagy initiation triggers p150Glued–AP-2β interaction on the lysosomes and facilitates their transport
Source: Cell Mol Life Sci. 2024 May 17;81(1):218. doi: 10.1007/s00018-024-05256-6 (PMC11101406; doi:10.1007/s00018-024-05256-6)
Supplement: Supplementary file 21 — Supplementary file21 (PDF 3616 KB) [file 18_2024_5256_MOESM21_ESM.pdf]

Supplementary Information

Autophagy initiation triggers p150^{Glued}-AP-2β interaction on the lysosomes and facilitates their transport.

Aleksandra Tempes, Karolina Bogusz, Agnieszka Brzozowska, Jan Weslawski, Matylda Macias, Oliver Tkaczyk, Katarzyna Orzoł, Aleksandra Lew, Malgorzata Calka-Kresa, Tytus Bernas, Andrzej A. Szczepankiewicz, Magdalena Mlostek, Shiwani Kumari, Ewa Liszewska,[1] Katarzyna Machnicka,[1] Magdalena Bakun, Tymon Rubel, Anna R. Malik, and Jacek Jaworski

Supplementary Materials and Methods

Plasmids

β-actin-GFP-p150^{Glued} and β-actin-tdTomato-p150^{Glued} were obtained by polymerase chain reaction (PCR)-based subcloning of the p150^{Glued} coding sequence into SalI/NotI sites of β-actin-GFP and β-actin-tdTomato, respectively. The pAvi-tag-thrombin-HA-p150^{Glued}N plasmid that encoded a fragment of p150^{Glued} (aa 1-490) was obtained by PCR using the following primers: 5’-GAAGAATTCATGGCCCAGAGCAAGAGGAC-3’ and 5’-GCGGGCGGCCGCTTAGGCGGCTTCCACTCGCTTCTG-3’, with pEGFPC2-Avi-tag-p150^{Glued} as a template. The resulting product was inserted into EcoRI/NotI restriction sites of pAvi-tag-thrombin-HA. pAvi-tag-thrombin-HA-p150^{Glued}C plasmid that encoded a fragment of p150^{Glued} (aa 490-1278) was generated analogically using the following primers: 5’-GAAGAATTCATGGCAGGCGCCCGAGTAAGG-3’ and 5’-GCGGGCGGCCGCTTAGGAGATGAGACGACCGTGAAG-3’. The pAvi-tag-thrombin-HA-p150^{Glued}C2 plasmid that encoded a fragment of p150^{Glued} (aa 1049-1278) was generated using 5’-GAAGAATTCATGGGCACTCCTGGGCAGGCTCCAGGCGC-3’ and 5’-GCGGGCGGCCGCTTAGGAGATGAGACGACCGTGAAG-3’ primers, with pAvi-tag-

thrombin-HA-p150^{Glued}C as a template. The resulting product was inserted into EcoRI/NotI restriction sites of pAvi-tag-thrombin-HA. The GST-p150^{Glued}C plasmid that encoded a fragment of p150^{Glued} (aa 490-1278) with a GST tag was generated by PCR from the HA-Avi-tag-p150^{Glued}C plasmid using 5'-GAAGAATTCATGGCAGGCGCCCGAGTAAGG-3' and 5'-GCGGGCGGCCGCTTAGGAGATGAGACGACCGTGAAG-3' primers. The product was inserted in pGEX-4T1 into EcoRI/NotI restriction sites. The GST-p150^{Glued}C2 plasmid that encoded a fragment of p150^{Glued} (aa 1049-1278) with a GST tag was obtained by PCR analogically using 5'-GAAGAATTCATGGGCACTCCTGGGCAGGCTCCAGGCGC-3' and 5'-GCGGGCGGCCGCTTAGGAGATGAGACGACCGTGAAG-3' primers. Plasmid encoding mCherry-AP-2β was obtained by exchanging GFP to mCherry in pEGFPC1-Ap2b1.

Antibodies

Table S1. Primary antibodies used for the study.

Primary antibody	Manufacturer, catalog no.	Application, dilution
mouse anti-p150^{Glued}	BD Bioscience, 610474	PLA 1:100<br>WB 1:1000<br>IF 1:200<br>IP 2 μg/reaction
rabbit anti-p150^{Glued}	Cell Signaling Technology, 69399	WB 1:1000
rabbit anti-AP-2β	Thermo Fisher, PA1-1066	PLA 1:100<br>IP 2 μg/reaction<br>IF 1:250
rabbit anti-AP-2β	Santa Cruz Biotechnology, sc-10762	WB 1:500
mouse anti-AP-2β	Santa Cruz Biotechnology, sc-74423	WB 1:500
goat anti-AP-2β	Santa Cruz Biotechnology, sc-6425	IF 1:200<br>IP 10 μg/reaction
mouse anti-AP-2α	BD Bioscience, 610502	WB 1:500
mouse anti-Arp1	Santa Cruz Biotechnology, sc-376010	WB 1:250
mouse anti-p62	Santa Cruz Biotechnology, sc-55603	WB 1:250
mouse anti-DIC1/2	Santa Cruz Biotechnology, sc-13524	WB 1:250

Rabbit anti-phospho-S6 Ser235/236	Cell Signaling Technology, 4858	WB 1:1000<br>IF 1:200
rabbit anti-S6	Cell Signaling Technology, 2217	IF 1:200
mouse anti-S6	Santa Cruz Biotechnology, sc-74459	WB 1:1000
mouse anti-CLIP-170	San<br>ta Cruz Biotechnology, 28325	WB 1:500<br>IF 1:500
rabbit anti-GFP	MBP, 598	WB 1:1000
mouse anti-tubulin	Sigma-Aldrich, T5168	WB 1:5000<br>IF 1:500
rabbit anti-tubulin	Abcam, ab4074	WB 1:3000
rabbit anti-LC3B	Sigma-Aldrich, L7543	WB 1:2500<br>IF 1:200
rabbit anti-LC3B	Cell Signaling Technology, 2775	IF 1:250
mouse anti-Lamp1	DSHB, H4A3	IF 1:100
mouse anti-beclin-1/BECN1	Santa Cruz Biotechnology, sc-48341	WB 1:250
rabbit anti-phospho-beclin-1 Ser30	Cell Signaling Technology, 54101	WB 1:500
rabbit anti-p62/SQSTM1	Cell Signaling Technology, 5114	WB 1:500<br>IF 1:250
mouse anti-Atg5	Cell Signaling Technology, 12994	WB: 1:500
mouse anti-actin	Sigma-Aldrich, A4700	WB 1:1000
mouse anti-GAPDH	Sigma-Aldrich, MAB374	WB 1:500
rabbit IgG	Cell Signaling Technology, 3900	IP 2 µg/reaction
goat IgG	Sigma-Aldrich, I5256-10MG	IP 10 µg/reaction
rabbit anti-neurofascin	Antibodies Incorporated, 75-172	Live imaging 1:100
mouse anti-his-tag	Cell Signaling Technology, 2366	WB 1:1000
rat anti-GST-tag	Chromotek, 6g9-100	WB 1:1000
rabbit anti-HA-tag	Cell Signaling Technology, 3724	WB 1:1000

PLA, proximity ligation assay; WB, Western blot; IF, immunofluorescence; IP, immunoprecipitation.

Cell line cultures and transfection

Rat2 and HEK293T cells were purchased from the American Type Culture Collection

and grown in Dulbecco's modified Eagle's medium (DMEM) with 4500 mg/ml of D-glucose that contained 10% fetal bovine serum (FBS) and 1% penicillin-streptomycin (all from Sigma-Aldrich) at 37°C in a 5% CO_2 atmosphere. For the proximity ligation assay (PLA) and PLA-electron microscopy (EM) experiments, Rat2 cells were grown on glass coverslips or Thermanox plastic coverslips (Thermo Fisher; catalog no. 174950), respectively, coated with 0.2% gelatin for 1 h at 37°C. For live experiments, Rat2 cells were seeded on glass coverslips that were coated with poly-L-lysine (50 μg/ml in H_2O for 1 h). For plasmid DNA transfection, HEK293T cells at 70% confluency were transfected using polyethylenimine PEI 25K (Polysciences, catalog no. 23966) according to the manufacturer's protocols. After transfection, HEK293T cells were grown in DMEM that was supplemented with 5% FBS for 48 h. Rat2 cells were transfected with plasmid DNA using electroporation. For each transfection, a total of 10^6 cells were suspended in Opti-MEM medium (Thermo Fisher, catalog no. 31985-047), mixed with 10 μg of DNA, and added to 2 mm gap cuvettes (Nepagene, catalog no. EC-002S). Cells were electroporated using a NEPA21 electroporator (Nepagene) with poring pulses (6×, 150 V, 2.5 ms length, 50 ms interval, 10% decay rate), followed by transfer pulses (5×, 20 V, 50 ms length, 50 ms interval, five pulses, 40% decay rate) with "±" polarity set for both. After electroporation, the cells were grown in DMEM with 10% FBS without antibiotics. The medium was changed the next day for a medium that contained 1% penicillin-streptomycin. The siRNA transfection of HEK293T and Rat2 cells was performed on trypsinized cells using Lipofectamine RNAiMAX Transfection Reagent (Thermo Fisher, catalog no. 13778150) according to the manufacturer's protocol. For the combined siRNA transfection/DNA electroporation experiments, cells after siRNA transfection were seeded on 10 cm plates and allowed to attach. After 24 h, cells were harvested by trypsinization and electroporated as described earlier. After electroporation, each variant was seeded on glass coverslips that were coated with poly-L-lysine (50 μg/ml in H_2O for 1 h).

Primary neuron preparation and transfection

Primary hippocampal cultures were prepared from embryonic day 18 rat brains as described previously [1]. The rats that were used to obtain neurons for further experiments were sacrificed according to protocols that complied with European Community Council Directive 2010/63/EU. Transfections of hippocampal neurons were performed on day *in vitro* 5 (DIV5) with Lipofectamine2000 (Thermo Fisher, catalog no. 11668019) as described previously [1], except that the incubation time with the transfection mixture was reduced to 2 h. DNA (2 μg) and 1.5 μl of Lipofectamine2000 were used per well of a 12-well dish.

Proximity ligation assay

For the PLA, cells were fixed for 5 min with ice-cold 100% methanol and 2 mM ethylene glycol-bis(β-aminoethyl ether)-*N*,*N*,*N'*,*N'*-tetraacetic acid (EGTA) at -20°C, followed by 10 min with 4% paraformaldehyde (PFA)/4% sucrose in phosphate-buffer (pH 7.4). Fixed cells were washed three times with phosphate-buffered saline (PBS) and incubated with mouse anti-p150^{Glued} and rabbit anti-AP-2β antibodies that were diluted in PBS that contained 1% donkey serum and 0.2% Triton-X100 at 4°C overnight. The next day, the cells were washed twice for 5 min with PBS with 1% donkey serum and 0.2% Triton-X100, washed once for 1 min in antibody diluent that was provided in the manufacturer's PLA kit (Sigma-Aldrich, catalog no. DUO92101), and then incubated for 60 min at 37°C with relevant secondary antibodies that were conjugated to oligonucleotide PLUS or MINUS (Sigma-Aldrich; catalog no. DUO92002 and DUO92004, respectively) and diluted in antibody diluent. The coverslips were then washed twice for 5 min with buffer A (Sigma-Aldrich, catalog no. DUO82046). Ligation and amplification were performed according to the manufacturer's protocol using Duolink In Situ Detection Reagents Red (Sigma-Aldrich, catalog no. DUO92008). The cells

were then counterstained with mouse anti-tubulin antibody that was diluted in PBS that contained 1% donkey serum and 0.2% Triton-X100 for 30 min at room temperature and washed three times with PBS and incubated with anti-mouse Alexa-488-conjugated secondary antibody that was diluted in PBS that contained 1% donkey serum and 0.2% Triton-X100 for 30 min at room temperature to visualize cell shape. Finally, the cells were washed three times with PBS and mounted with DuoLink *in situ* mounting medium that contained DAPI (Sigma-Aldrich, catalog no. DUO82040). For each experiment, negative controls that lacked one of the primary antibodies were used.

Immunofluorescence

For analyses of the co-localization of p150^{Glued} and AP-2β proteins, Rat2 cells were fixed with ice-cold 100% methanol with 2 mM EGTA for 5 min at -20°C and then with 4% PFA/4% sucrose in phosphate-buffer (pH 7.4) for 10 min at room temperature and washed three times with PBS. After fixation, the cells were blocked for 1 h in blocking buffer (5% donkey serum, 0.3% Triton X-1001 in PBS). Next, the cells were incubated overnight with primary antibodies in antibody dilution buffer (1% BSA, 0.3% Triton X-100 in PBS) at 4°C and then washed three times with PBS at room temperature. Afterward, specimens were incubated with Alexa 488- and Alexa 594-conjugated secondary antibodies for 1 h at room temperature, followed by three washes with PBS. Coverslips were mounted with Prolong Gold with DAPI (Thermo Fisher, catalog no. P36941). For the immunofluorescent staining of ribosomal protein S6 phosphorylated at Ser235/236 (P-S6), neurons were fixed with 4% PFA/4% sucrose in phosphate-buffer (pH 7.4) for 10 min at room temperature and washed three times with PBS. After fixation, the cells were blocked for 1 h in blocking buffer (5% donkey serum, 0.3% Triton X-1001 in PBS). Next, the cells were incubated overnight with primary antibody in antibody dilution buffer I (1% BSA, 0.3% Triton X-100 in PBS) at 4°C and then washed three times with

PBS at room temperature. Alexa 488-conjugated secondary antibody with Alexa 647-conjugated phalloidin (1:1000; Thermo Fisher Scientific) in antibody dilution buffer was next added for 1 h at room temperature. Afterward, the cells were washed three times with PBS, and coverslips were mounted with Prolong Gold (Thermo Fisher, catalog no. P36934). For the immunofluorescent detection of CLIP-170, the cells were fixed for 5 min with ice-cold 100% methanol and 2 mM EGTA at -20°C, followed by 10 min with 4% PFA/4% sucrose in phosphate-buffer (pH 7.4) at room temperature, and then washed three times with PBS. Next, the cells were washed twice with antibody dilution buffer II (1% donkey serum, 0.2% Triton X-100 in PBS) and incubated overnight with the primary antibody in antibody dilution buffer II at 4°C. After incubation with primary antibodies, the cells were washed three times with PBS at room temperature and incubated with Alexa 488-conjugated secondary antibody in antibody dilution buffer II for 1 h at room temperature. Afterward, the cells were washed three times with PBS, and nuclei were stained with Hoechst 33258 (1 μg/ml; Thermo Fisher Scientific) for 5 min. The cells were then washed two more times with PBS, and coverslips were mounted with Prolong Gold. To analyze LC3B immunofluorescence, the cells were fixed according to the CLIP-170 staining protocol. After fixation, the cells were blocked for 15 min at room temperature in antibody dilution buffer III (2% donkey serum, 0.2% Triton X-100 in PBS) and incubated for 1 h with the primary antibody in antibody dilution buffer III at room temperature. The cells were then washed three times with antibody dilution buffer III at room temperature and incubated with Alexa 488-conjugated secondary antibody in antibody dilution buffer III for 1 h at room temperature. The next steps were identical to the description of CLIP-170 immunofluorescence.

Fixed cell image acquisition and analysis

Microscopic images of fluorescently labeled *in vitro* cultured cells, with the exception

of quantitative co-localization experiments, were acquired using a Zeiss LSM800 confocal microscope (40× or 63× Plan Apo oil immersion objective, NA=1.4) as Z-stacks at 0.2-0.5 μm intervals at 1024 × 1024 pixel resolution. To obtain higher image resolution and conduct studies of protein co-localization, an AiryScan detector was used with the 63× oil objective. To enable comparisons between images, the microscope settings were kept constant for all scans. The Z-stacks, with the exception of images acquired in AiryScan mode, were converted to single images using a maximum intensity projection. For quantification of the number of PLA puncta in Rat2 cells, the cell shape was indicated as a region of interest for each cell, and the threshold was set manually and uniformly for all images in each experiment to extract specific signals from background. The number of PLA puncta per cell was counted in ImageJ software using the "Analyze Particles" macro. The average number of PLA puncta per cell was then calculated for each condition, and the results were normalized by subtracting the average number of PLA puncta per cell from the negative control. The obtained average number of PLA puncta per cell was further normalized by dividing the obtained values from a particular experiment by the average number of PLA puncta per cell from the control from at least three repetitions. For the analysis of P-S6 levels in neurons, the mean fluorescent intensity of P-S6 was measured in the neuronal soma using ImageJ software.

For quantitative co-localization analysis, images of fluorescently labeled cells were registered using a Leica SP8 confocal microscope with a 63× oil immersion Plan Apo objective lens (NA=1.4). The microscope system was equipped with acousto-optical beam splitter and multialkali single-channel PMTs and hybrid detectors (HyD). The fluorescence of DAPI was excited with a 405 nm light (5 mW diode laser) and detected in the 410-470 nm range. The fluorescence of Alexa 488 and Alexa 568 was excited with a supercontinuum (white light) laser (15 mW) at their absorption maxima and detected at 490-550 nm and 575-630 nm ranges, respectively. The images (optical sections) were registered using PMT (gain 600, DAPI) or

HyD (gain 200, Alexa) working in the integration mode at 16-bit precision. The pixel size was 90 nm in the x-y plane and 350 nm along the optical axis. The pixel dwell time was 2.5 µs (4× averaging), and the confocal pinhole was set to 1 Airy unit (at 530 nm). The cells were imaged at room temperature. Stacks of optical sections (Alexa 488 and Alexa 568) were subjected to blind deconvolution (15 iterations) using Huyghens v. 3.7 (Scientific Volume Imaging, Hilversum, The Netherlands). Processing was initialized with the nominal PSF of the objective and performed at an SNR set to 15. The background was estimated as a minimum of average intensity in a 25 × 25 region. Following deconvolution, the nuclei (DAPI) were segmented using Otsu thresholding (two classes). Overlapping nuclei were separated by Euclidean distance transform, followed by watershedding. The Alexa 488 and Alexa 568 images were summed, processed with a median filter (7 × 7 × 3 size) and dilated (11 × 11 × 3 neighborhood). The cells were segmented using Otsu thresholding, and single cell masks were constructed with iterative dilation of the nuclear masks. The dilation steps were ordered by the intensity of processed cell images. Volumes that corresponded to nuclei were then excluded from the cell masks. Coefficients of intensity correlation (Pearson and Spearman) between Alexa 488 and Alexa 568 images were calculated on a cell-by-cell basis using the respective masks. The operation was repeated using the Alexa 568 images that were shifted by ±8 voxels in the x and y directions and ±2 voxels in the z direction. The respective coefficients were calculated using the union of two respective cellular masks and corresponded to the random association of Alexa 488 and Alexa 568 fluorescence in a cell. The raw correlation coefficients were then divided by their random counterparts (on a cell-by-cell basis) to create standardized Pearson and Spearman values.

Analysis of PLA signal co-occurrence with Lamp-1-GFP

Analysis of the proximity of the PLA dots to the LAMP1-GFP vesicles was performed

using 16-bit microscopic images acquired with the LSM800 Airyscan module. Only the middle optical section from 5-step z-stack was used for the analysis. The analysis was performed with the ImageJ software. First, the signal of a channel containing PLA puncta was converted into a binary mask using a manually selected threshold for each photo. Then, the captured binary points were expanded using the “Dilate” command to enable the detection of events where the signals touch but do not directly overlap. Based on these points, regions of interest (ROIs) were created in which the fluorescent signal of the GFP channel was measured using the mean gray value. Finally, calculations were performed in which points were labeled as co-occurring with Lamp1-GFP if the mean fluorescence signal of GFP in the ROI was above the established threshold of 1000 pixel brightness value.

Live imaging of microtubule dynamics

For microtubule dynamics analysis, Rat2 cells were electroporated with pEGFP-EB3 or pEGFP-CLIP-170 plasmids. Forty-eight hours later, time-lapse movies of EGFP-EB3 or EGFP-CLIP-170 comets were taken using an Andor Revolutions XD spinning disc microscope with a 63x objective and 1.6 x tube lens (optovar) at 1004 x 1002 pixel resolution. Images were taken with an exposure of 200 ms and interval of 0.3 s, collecting a total of 600 frames over 3 min. During imaging, cells were kept in a Chamlide magnetic chamber (Quorum Technologies) at 37°C with 5% CO2 in the incubator that was part of the microscope system. Only EGFP-EB3 or EGFP-CLIP-170 comets whose movements lasted at least four consecutive frames and had a displacement length of at least 10 pixels (0.68 μm) were analyzed using the ImageJ “TrackMate” plugin. The reported values are the number of tracks (i.e., the quantification of objects that were detected as comets), the total run length of comets before catastrophe (Track Displacement), comet lifetime (Track Duration), and Track Mean Speed. For the analysis, values were calculated as means for each cell.

Opera Phenix High content imaging of fixed cells

For the high-throughput analysis of effects of different drugs on lysosomal acidity, Rat2 cells were seeded on CellCarrier-96 well Black glass bottom plates (Perkin Elmer) that were coated with 0.2% gelatin at a density of 7×10^3 cells per well 1 day before treatment (see above). After 80 min of drug exposure, LysoTracker Red DND-99 (500 nM, Invitrogen, catalog no. L7528) was added to the cells for the last 40 min. Subsequently, the cells were fixed with 4% PFA for 10 min and subjected to three 10-min washes with PBS. High-content screening microscopy was conducted using the Opera Phenix system (PerkinElmer) that was equipped with a 40× 1.1 NA water immersion objective. Image acquisition and subsequent analysis were performed using Harmony 4.9 software (PerkinElmer). Statistical analyses were performed using the R and RStudio software packages (Cran). To ensure consistent and reliable data analysis, cells were initially filtered based on morphological criteria to obtain uniform cell populations. The identification of lysotracker-positive compartments was achieved using the "Find spots" algorithm that is integrated within the Harmony software. Additionally, the total signal intensity of Lysotracker staining was quantified within both the cell cytoplasm and the previously defined compartment regions.

Protein extraction from adult rat brains and immunoprecipitation

For the isolation of endogenous proteins from brain extracts, Dynabeads™ Protein G resin conjugated to the primary antibody recognizing the selected protein was prepared. For this purpose, 30 μl of the resin was incubated with 1 μg of the antibody for 1 hour at room temperature. A non-specific IgG antibody from the same animal species as the respective primary antibody was used as a negative control. Rat cortex extract was obtained by homogenizing the tissue in lysis buffer containing 40 mM HEPES, pH 7.5, 150 mM NaCl, 1

mM $MgCl_2$, 0.5 mM $CaCl_2$, 0.3 % CHAPS, protease and phosphatase inhibitors. In case of rapamycin-treated variant, 100 nM rapamycin was added to the lysis buffer for brains of rapamycin-treated animals. The homogenate was incubated for 1 hour at 4 °C, and then centrifuged (15 minutes, 4 °C, 18,000 x *g*) to remove cell debris. The protein concentration in the filtrate was then determined using the Pierce BCA Protein Assay Kit (Thermo Fisher Scientific; catalog no. 23225). A portion of the clear protein extract at 3 mg/ml was retained as the starting fraction ("input"), and the remainder was applied to antibody-conjugated beds and incubated overnight at 4 °C. The next day, the beds were washed four times with a wash buffer consisting of 40 mM HEPES, pH 7.5; 300 mM NaCl; 1 mM $MgCl_2$; 0.5 mM $CaCl_2$; 0.1 % CHAPS. After the last wash, the bed with the bound proteins was resuspended in Laemmli buffer and heated at 96 °C for 5 minutes. The prepared samples were analyzed using the Western blot technique.

Protein production in bacteria and pull-down experiment

Recombinant proteins were produced in the *E. coli* BL21 strain. Individual clones that were transformed with plasmids that encoded proteins of interest were picked from the plates and inoculated to 5 ml of LB with appropriate antibiotic. After overnight culture, at 37°C with shaking, bacteria were refreshed with new medium in a 1:50 ratio and further cultured until reaching an optical density at 600 nm (OD_{600}) of 0.6-0.8. Protein production was induced using 1 mM isopropyl-β-D-1-thiogalactopyranoside (Carl Roth, catalog no. CN08.3). The His_6-AP-2β appendage domain, GST-Eps15, and free GST-tag were produced at 37°C for 2 h, and GST-$p150^{Glued}$ fragments were produced at 21°C overnight. For protein purification, cultures were centrifuged at 4,500 × *g* for 10 min, and the pellet was resuspended in lysis buffer (50 mM Tris [pH, 8.0], 150 mM NaCl, 0.1% Triton X-100, and protease inhibitors). For all subsequent stages, lysates were kept on ice. After resuspension, the cells were lysed by sonication in a

Sonics VCX130 PB sonicator (Vibra-Cell) in two 30-s sessions with a 70% amplitude. After sonication, lysates were centrifuged at 13,000 × *g* for 5 min to remove the insoluble fraction. For the pull-down experiment, the bait protein was then added to Glutathione-Sepharose 4B resin (Merck, catalog no. GE17-0756-01) at a ratio of 30 μl beads for each 10 ml of original bacterial culture and incubated for 1 h with end-to-end rotation. After incubation, the resin was washed three times with lysis buffer, and the prey protein lysate was added. After another 1 h of incubation, the resin was again washed three times with lysis buffer and prepared for Western blot by dissolving in 1× Laemmli buffer and incubation in a heat block at 94°C for 15 min.

Whole-cell lysate preparation

For the Western blot analysis of proteins in whole-cell lysates, Rat-2 cells were lysed in lysis buffer (20 mM Tris [pH 7.5], 150 mM NaCl, 2 mM ethylenediamine tetraacetic acid [EDTA], 0.5% Triton X-100, 0.5% NP-40, 2 mM $MgCl_2$, and 10% glycerol supplemented with protease and phosphatase inhibitors). The total protein concentration in whole-cell lysates was measured using the Pierce BCA Protein Assay Kit (Thermo Fisher Scientific) according to the manufacturer's instructions. Afterward, 4× Laemmli sample buffer was added to the lysates, followed by boiling for 5 min at 95°C.

Immunoprecipitation

For the immunoprecipitation (IP) of endogenous proteins from HEK293T cells, the cells were lysed for 15 min on ice in lysis buffer (20 mM HEPES [pH 7.5], 120 mM KCl, and 0.3% 3-[(3-cholamidopropyl)dimethylammonio]-1-propanesulfonate [CHAPS] supplemented with protease and phosphatase inhibitors, in addition to 100 nM rapamycin for variants in which cells were treated with rapamycin) and spun. Next, 2 μg of anti-AP-2β or anti-p150^{Glued} primary antibody or IgG (as a negative control) was added to the supernatant and incubated overnight

at 4°C while rotating. The next day, a mixture of lysate and antibodies was added to 30 µl of Dynabeads Protein G (Thermo Fisher, catalog no. 10004D) and incubated for 4 h at 4°C while rotating. Beads were then washed four times in wash buffer (20 mM HEPES [pH 7.5], 120 mM KCl, and 0.1% CHAPS), eluted in 2× Laemmli sample buffer, incubated for 5 min in 95°C and analyzed by sodium dodecyl sulfate-polyacrylamide gel electrophoresis (SDS-PAGE), followed by Western blot. The IP of AP-2β from rat brain extracts was performed as described previously [2]. For the IP of heterologous proteins, HEK293T cells were transfected with pEGFPC1-Ap2b1, GFP-β-actin-p150^{Glued}, or pEGFPC1 (as a negative control). Forty-eight hours after transfection, the cells were lysed for 15 min on ice in lysis buffer (10 mM Tris [pH 7.5], 150 mM NaCl, 0.5 mM EDTA, and 0.15 % CHAPS supplemented with protease and phosphatase inhibitors) and spun using a benchtop centrifuge at maximum speed for 15 min at 4°C. The lysates were then incubated with GFP-Trap Agarose (20 µl per variant; Chromotek, catalog no. gta-10) for 2 h at room temperature, followed by four washes with wash buffer (10 mM Tris [pH 7.5], 150 mM NaCl, and 0.5 mM EDTA). The immunoprecipitated proteins were next used for the mTOR kinase assay.

Avi-tag pull down of biotinylated proteins and AP-2β-ear binding

The His_6-AP-2β appendage domain was produced as described in the *Protein production in bacteria* section. For protein purification, cultures were centrifuged at 4,500 × *g* for 10 min, and the pellet was resuspended in lysis buffer (50 mM NaH_2PO_4/Na_2HPO_4 [pH 8], 300 mM NaCl, 10 mM imidazole, 10% glycerol, 10 mM β-mercaptoethanol, 0.1% Triton X-100, and protease inhibitors). For all subsequent stages, lysates were kept on ice. After resuspension, the cells were lysed by sonication in a Sonics VCX130 PB sonicator (Vibra-Cell) in two 25-s sessions with a 70% amplitude. After sonication, the lysates were centrifuged at 13,000 × *g* for 5 min to remove the insoluble fraction. The column with 5 ml agarose - Ni -

NTA used for affinity chromatography of recombinant protein was equilibrated with 20 ml of lysis buffer and a flow rate of approximately 1 ml/min. The cell lysate that was obtained from 2 L of culture was then added to the column at a flow rate of approximately 0.5 ml/min. After the lysate passed through the column, the agarose resin was washed successively with 50 ml of appropriate buffers: lysis buffer, lysis buffer supplemented with 2 M NaCl, and lysis buffer supplemented with 20 mM imidazole. The protein was then eluted with 25 ml of elution buffer (50 mM NaH_2PO_4/Na_2HPO_4 [pH 8], 2 M NaCl, 250 mM imidazole, 10% glycerol, 10 mM β-mercaptoethanol 0.1% Triton X-100). One milliliter fractions were collected and verified for the presence of purified protein. Those that contained the highest amount of the His_6-AP-2β ear domain were pooled and used for the binding experiments. M-280 streptavidin Dynabeads (Thermo Fisher, catalog no. 11205D) were incubated for 1 h with blocking buffer (20 mM HEPES [pH 7.5], 120 mM KCl, 0.5 mg/ml BSA, and 20% glycerol). The beads were washed with lysis buffer (20 mM HEPES [pH 7.5], 120 mM KCl, and 0.3% CHAPS supplemented with protease and phosphatase inhibitors). Forty-eight hours after transfection with BirA and plasmids that encoded Avi-tagged proteins, HEK293T cells were lysed for 15 min on ice in lysis buffer and spun using a benchtop centrifuge at maximum speed (15 min, 4°C). The supernatant was added to the previously prepared M-280 streptavidin Dynabeads and incubated together for 1 h at 4°C while rotating. The beads were then washed on ice four times in wash buffer (20 mM HEPES [pH 7.5], 500 mM KCl, and 0.1% CHAPS) and incubated with 0.2 mg/ml His_6-AP-2β ear domain purified from bacteria in binding buffer (20 mM HEPES [pH 7.5], 300 mM KCl, and 0.1% CHAPS) for 2 h at 4°C while rotating. The beads were then washed four times with binding buffer, eluted in 2× Laemmli sample buffer, incubated for 5 min in 95°C and analyzed by SDS-PAGE, followed by Western blot.

Western blot

Protein samples were analyzed by SDS-PAGE and immunoblotting according to standard laboratory protocols. Primary antibodies that were used for Western blot are listed in Table 1. Proteins of interest were detected using HRP- or IRdye-conjugated secondary antibodies in a chemo-luminescence reaction or with the Odyssey LiCor Biosciences system, respectively. The densitometry analysis of the amount of LC3B was performed using Image Studio Lite (LiCor Biosciences) and a previously described method [3]. Specifically, the signal intensities for LC3B I and LC3B II were first measured. Based on these values, normalization was performed. First, for all variants in a single replicate, the sum of intensities for a given protein was calculated. Second, individual protein band intensities were divided by this sum. Such normalization was performed for LC3B I and LC3B II. Finally, after normalizing the values, the ratio between LC3B II and LC3B I was calculated by dividing the normalized values of LC3B II by LC3B I.

Kinase assays

An *in vitro* phosphorylation assay was performed for 20 min at 30°C in kinase assay reaction buffer (25 mM HEPES [pH 7.5], 50 mM KCl, and 10 mM $MgCl_2$) in the presence of 2.5 μCi [γ-^{33}P]ATP (Hartmann Analytic) and 2.5 mM "cold" ATP using a recombinant, active mTOR kinase fragment (1362-2549 aa; Millipore, catalog no. 14-770) and proteins that were immunoprecipitated from HEK293T cells or commercial mTOR substrate (Merck Millipore, catalog no. 12-645). The reaction was stopped by adding 4× Laemmli sample buffer and boiling for 5 min at 95°C. Next, the samples were separated by SDS-PAGE. The gel was stained with Coomassie Brilliant Blue and dried. The radioactive signal was detected and analyzed using a Typhoon Trio+ Phosphorimager (GE Healthcare).

RNA Isolation for Quantitative Real-Time PCR

RNA was isolated from Rat2 cells using the RNeasy Mini Kit (Qiagen) and reverse transcribed with the High-Capacity cDNA Reverse Transcription Kit (Thermo Fisher Scientific). Quantitative real-time polymerase chain reaction (qRT-PCR) was performed using FAM fluorophore TaqMan probes with TaqMan Gene Expression Master Mix buffer (Applied Biosystems). TaqMan probes used for the experiments were the following: tubulin (Rn01532518_g1) and Snap29 (Rn00587482_m1; Thermo Fisher). Data were acquired with the LightCycler 96 System (Roche) and analyzed using the $2^{(-\Delta\Delta Ct)}$ method for relative quantification.

Supplementary Methods References

1. Swiech L, Blazejczyk M, Urbanska M, Pietruszka P, Dortland BR, Malik AR, Wulf PS, Hoogenraad CC, Jaworski J (2011) CLIP-170 and IQGAP1 Cooperatively Regulate Dendrite Morphology. J Neurosci 31:4555–68. https://doi.org/10.1523/JNEUROSCI.6582-10.2011

2. Kononenko NL, Claßen GA, Kuijpers M, Puchkov D, Maritzen T, Tempes A, Malik AR, Skalecka A, Bera S, Jaworski J, Haucke V (2017) Retrograde transport of TrkB-containing autophagosomes via the adaptor AP-2 mediates neuronal complexity and prevents neurodegeneration. Nat Commun 8:14819. https://doi.org/10.1038/ncomms14819

3. Degasperi A, Birtwistle MR, Volinsky N, Rauch J, Kolch W, Kholodenko BN (2014) Evaluating strategies to normalise biological replicates of Western blot data. PLoS One 9:e87293. https://doi.org/10.1371/journal.pone.0087293

Supplementary Figures

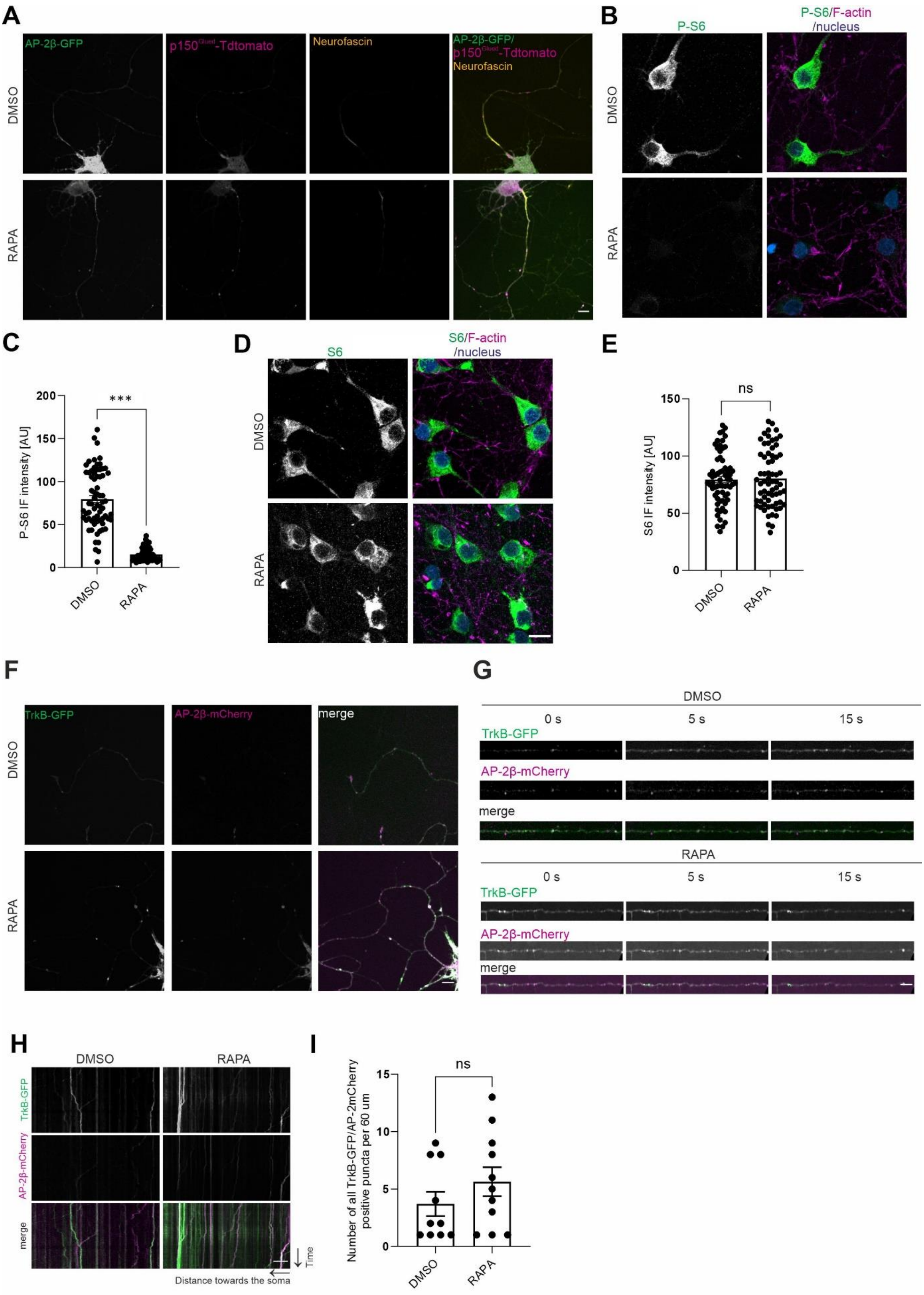

Fig. S1. mTORC1 inhibition in neurons increases p150^{Glued}–AP-2β and TrkB– AP-2β

interactions in axons. (**A**) Snapshots of DIV7 neurons that were treated as indicated and expressed p150^{Glued}-Tdtomato (magenta) and AP-2β-GFP (green) and stained for the axon initial segment with CF640R-conjugated neurofascin antibody (orange). Scale bar = 10 µm. (**B**) Representative pictures of DIV7 neurons that were treated as indicated and immunofluorescently labeled for endogenous P-S6 (Ser235/236) (green) and stained with Alexa Fluor 647 phalloidin (magenta). Scale bar = 10 µm. (**C**) Quantification of P-S6 (Ser235/236) immunofluorescence level in cell bodies of neurons that were treated as indicated. The data are expressed as the mean fluorescence intensity ± SEM. $N = 3$ independent experiments. $n = 69$ cells (DMSO), 69 cells (RAPA). ***$p < 0.001$ (Mann-Whitney test). (**D**) Representative pictures of DIV7 neurons that were treated as indicated and immunofluorescently labeled for endogenous S6 (green) and stained with Alexa Fluor 647 phalloidin (F-actin, magenta). Scale bar = 10 µm. (**E**) Quantification of S6 immunofluorescence level in cell bodies of neurons that were treated as indicated. The data are expressed as the mean fluorescence intensity ± SEM. $N = 3$ independent experiments. $n = 66$ cells (DMSO), 66 cells (RAPA). ***$p < 0.001$ (Mann-Whitney test). (**F**) Snapshots of DIV7 neurons that were treated as indicated and expressed TrkB-GFP (green) and AP-2β-mCherry (magenta). Scale bar = 10 µm. (**G**) Representative snapshots of 60 µm segment of axon and (**H**) corresponding kymographs of TrkB-GFP and AP-2β-mCherry-positive objects. See also Movies 5-8. Scale bar = 10 µm. (**I**) Number of all TrkB-GFP/AP-2β-mCherry objects in axons per 60 µm. The data are expressed as the mean ± SEM. $N = 4$ independent experiments. $n = 10$ cells (DMSO), 11 cells (RAPA); *ns*, nonsignificant (Mann-Whitney test).

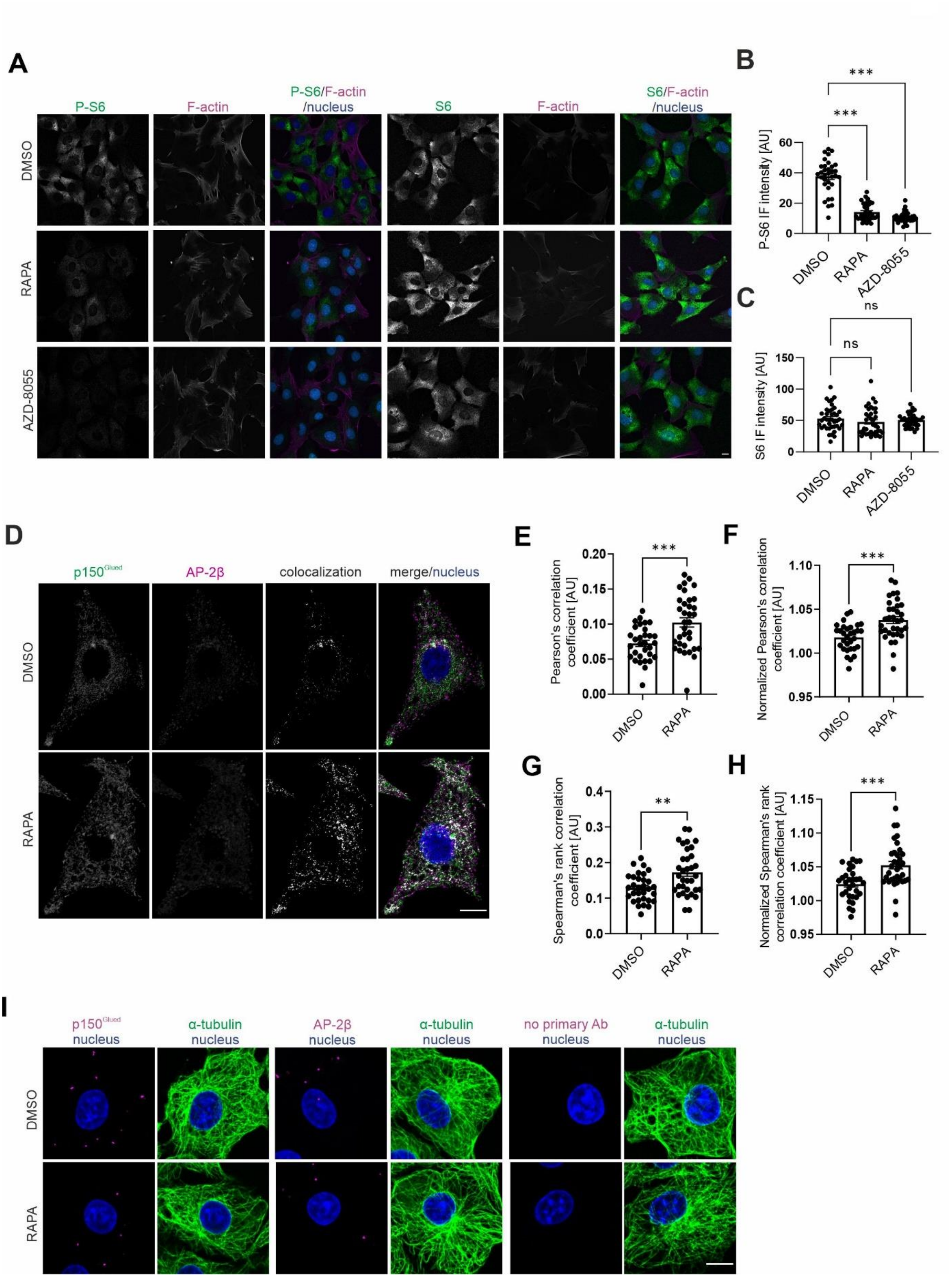

Fig. S2. mTORC1 inhibition increases p150^{Glued}–AP-2β interaction in non-neuronal cells. (**A**) Representative images of Rat2 cells that were treated for 2 h with 0.1% DMSO, 100 nM rapamycin (RAPA), or 100 nM AZD-8055 with immunofluorescently labeled endogenous P-

S6 (Ser235/236) or S6 (green), Alexa Fluor 647 phalloidin (F-actin; magenta) and nuclei that were stained with Hoechst 33258 (blue). Scale bar = 10 µm. (**B**) Quantification of P-S6 (Ser235/236) immunofluorescence level in Rat2 cells that were treated as in *A*. The data are expressed as the mean fluorescence intensity ± SEM. $N = 2$ independent experiments. $n = 33$ cells (DMSO), 35 cells (RAPA). 35 cells (AZD-8055). $^{***}p < 0.001$ (Kruskal-Wallis test followed by Dunn's *post hoc* test). (**C**) Quantification of S6 immunofluorescence level in Rat2 cells that were treated as in *A*. The data are expressed as the mean fluorescence intensity ± SEM. $N = 2$ independent experiments. $n = 40$ cells (DMSO), 38 cells (RAPA). 38 cells (AZD-8055). $^{***}p < 0.001$ (Kruskal-Wallis test followed by Dunn's *post hoc* test). (**D**) Representative images of Rat2 cells that were treated for 2 h with 0.1% DMSO or 100 nM rapamycin (RAPA) with immunofluorescently labeled endogenous $p150^{Glued}$ (green) and AP-2β (magenta) and additionally stained with DAPI (blue). Scale bar = 10 µm. The white channel indicates $p150^{Glued}$ and AP-2β immunofluorescent signal co-localization. (**E**) Analysis of Pearson's correlation coefficients of endogenous AP-2β/$p150^{Glued}$ co-localization without normalization. The data are expressed as the mean coefficient of co-localization of two proteins ± SEM. $N = 2$ independent experiments. $n = 31$ cells (DMSO), 34 cells (RAPA). $^{***}p < 0.001$ (Student's *t*-test). (**F**) Analysis of Pearson's correlation coefficients of AP-2β/$p150^{Glued}$ with normalization to the appropriate value that resulted from the random distribution of fluorescence in the cell. The data are expressed as the mean coefficient of co-localization of two proteins ± SEM. $N = 2$ independent experiments. $n = 31$ cells (DMSO), 34 cells (RAPA). $^{***}p < 0.001$ (Student's *t*-test). (**G**) Analysis of Spearman's rank correlation coefficient of AP-2β/$p150^{Glued}$ co-localization without normalization. The data are expressed as the mean coefficient of co-localization of two proteins ± SEM. $N = 2$ independent experiments. $n = 31$ cells (DMSO), 34 cells (RAPA). $^{**}p < 0.005$ (Student's *t*-test). (**H**) Analysis of Spearman's rank correlation coefficient of AP-2β/$p150^{Glued}$ co-localization with normalization to the appropriate value that

resulted from the random distribution of fluorescence in the cell. The data are expressed as the mean coefficient of co-localization of two proteins ± SEM. $N = 2$ independent experiments. $n = 31$ cells (DMSO), 34 cells (RAPA). $***p < 0.001$ (Student's t-test). (**I**) Representative images of Rat2 fibroblasts with negative controls for the PLA with one primary antibody present ($p150^{Glued}$ or AP-2β) or without primary antibodies (Ab) (magenta), with immunofluorescently labeled tubulin (green) and DAPI-stained nuclei (blue). Cells were treated for 2 h with 0.1% DMSO or 100 nM rapamycin (RAPA). Scale bar =10 µm.

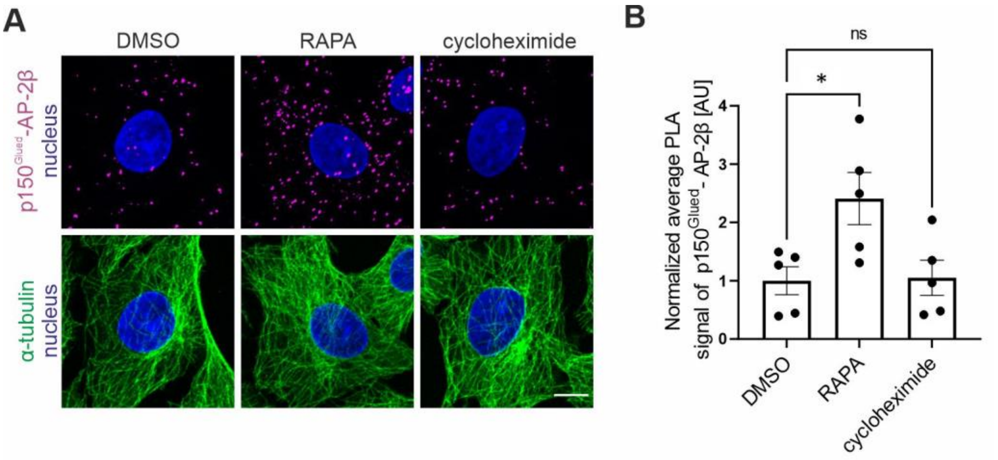

Fig. S3. Protein synthesis inhibition does not enhance $p150^{Glued}$–AP-2β interaction. (**A**) Representative images of Rat2 fibroblasts treated for 2 h with 0.1% DMSO, 100 nM rapamycin (RAPA), or 35 µM cycloheximide with $p150^{Glued}$–AP-2β PLA signals (magenta), immunofluorescently labeled tubulin (green) and DAPI-stained nuclei (blue). Scale bar = 10 µm. (**B**) Quantification of the number of $p150^{Glued}$–AP-2β PLA puncta in cells that were treated as in A. The data are expressed as the mean number of PLA puncta per cell, normalized to the control variant (DMSO) ± SEM. $N = 5$ independent experiments. $n = 173$ cells (DMSO), 210 cells (RAPA), 172 cells (cycloheximide). $*p < 0.05$, *ns* – nonsignificant (one-way ANOVA followed by Bonferroni *post hoc* test).

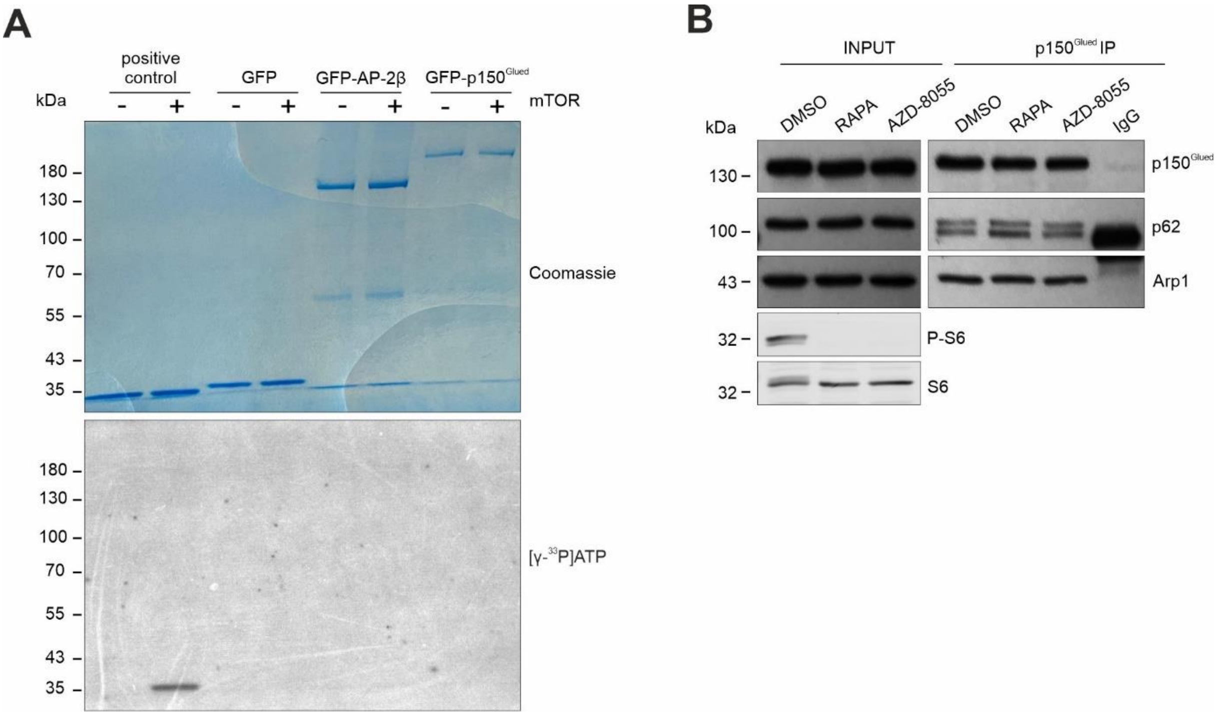

Fig. S4. mTOR does not phosphorylate AP-2β or p150^{Glued} and does not affect p150^{Glued} binding to Arp1 or p62. (**A**) Results of kinase assay using recombinant active mTOR fragment and GFP-AP-2β, GFP-p150^{Glued}, or GFP (negative control) immunoprecipitated from HEK293T cells as substrates. Commercial mTOR substrate served as a positive control. The upper panel shows the Coomassie staining of SDS-PAGE gels with the analyzed proteins. The lower panel shows the radioactive signal level of [γ-33P] ATP that was incorporated into the analyzed proteins. Shown is a representative example from $N = 2$ independent experiments. (**B**) Western blot analysis of endogenous p150^{Glued}, Arp1, p62, S6, and P-S6 (Ser235/236) levels in HEK293T cells and co-immunoprecipitation of p150^{Glued}, Arp1, and p62 from HEK293T cells that were treated for 2 h with 0.1% DMSO, 100 nM rapamycin (RAPA), or 100 nM AZD80550. Shown is a representative example from $N = 2$ independent experiments.

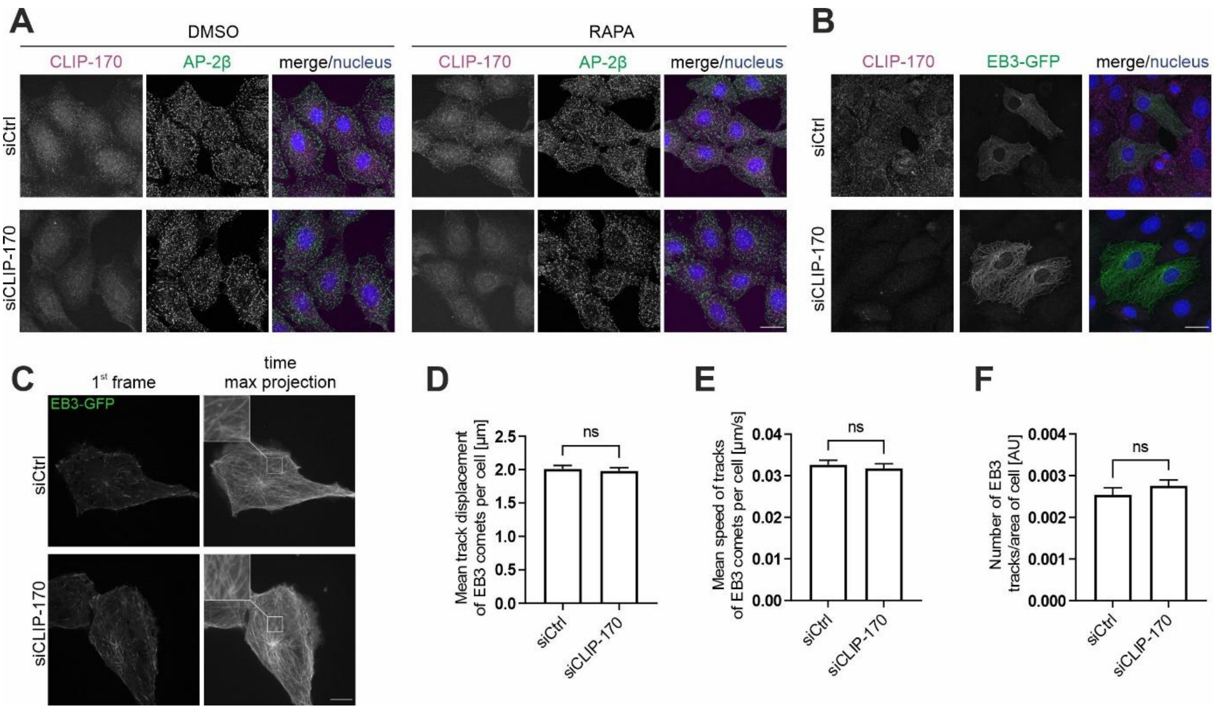

Fig. S5. CLIP-170 knockdown does not influence AP-2β distribution or dynamic microtubules. (**A**) Representative images of fluorescently labeled endogenous CLIP-170 (magenta), AP-2β (green), and nuclei stained with Hoechst 33258 (blue) in Rat2 cells that were transfected with siCtrl or rat siCLIP-170 for 72 h, followed by treatment with 0.1% DMSO or 100 nM rapamycin for 2 h. Scale bar = 20 µm. (**B**) Representative images of fluorescently labeled endogenous CLIP-170 (green) and nuclei stained with Hoechst 33258 (blue) in Rat2 cells that were transfected with siCtrl or rat siCLIP-170 and 24 h later electroporated with a plasmid that encoded EB3-GFP. Cells were fixed 48 h after electroporation. Scale bars = 20 µm. (**C**) Representative images from time-lapse movies of microtubule dynamics in Rat2 cells that were transfected with siCtrl or siCLIP-170 and 24 h later electroporated with a plasmid that encoded EB3-GFP. The experiment was performed 48 h after electroporation. Images in the left column show the first frame from the time-lapse movies. Images in the right column show maximum intensity projections from Z-stacks of all 600 frames. Scale bar = 10 µm. (**D**) Quantification of the mean track run lengths of EB3-GFP comets per cell. The data are expressed as the mean ± SEM. *N* = 3 independent experiments. *n* = 26 cells (siCtrl), 26 cells

(siCLIP-170). *ns*, nonsignificant (Student's *t*-test). (**E**) Quantification of the mean speed of EB3-GFP comet. The data are expressed as the mean ± SEM. *N* = 3 independent experiments. *n* = 26 cells (siCtrl), 26 cells (siCLIP-170). *ns*, nonsignificant (Mann-Whitney test). (**F**) Quantification of the number of all microtubule tracks per cell divided by cell area for EB3-GFP. The data are expressed as the mean ± SEM. *N* = 3 independent experiments. *n* = 26 cells (siCtrl), *n* = 26 cells (siCLIP-170). *ns*, nonsignificant (Student's *t*-test).

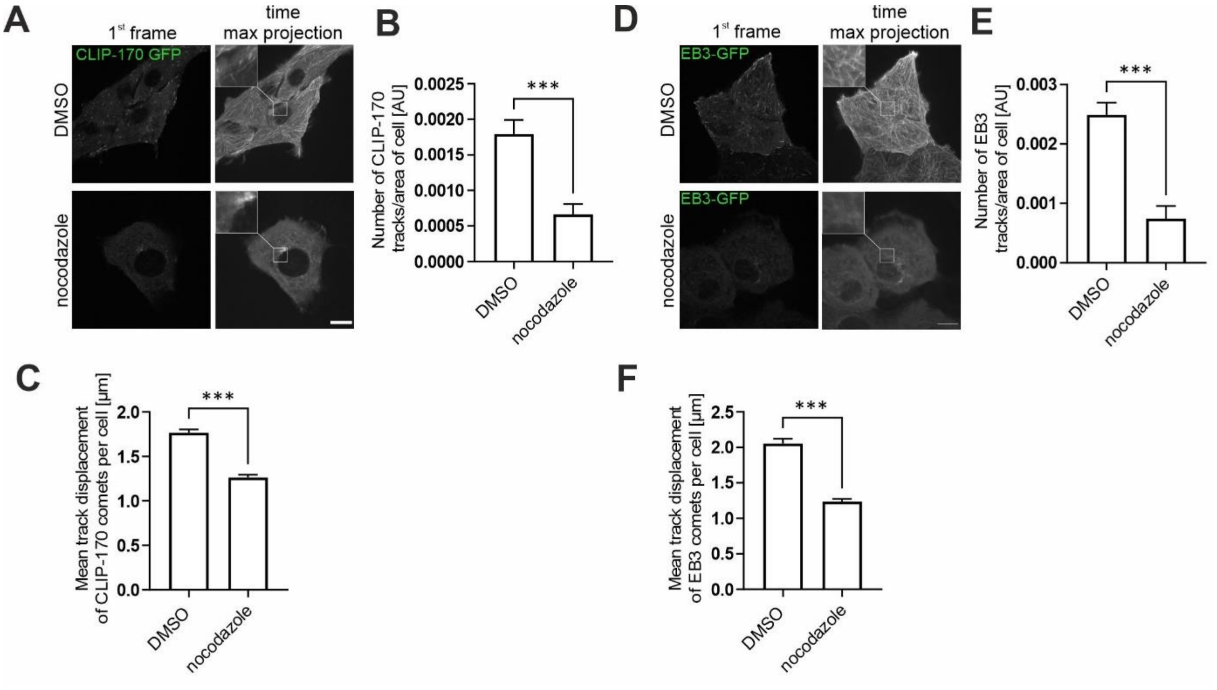

Fig. S6. Low doses of nocodazole affect microtubule dynamics. (**A**) Representative images from time-lapse movies of microtubule dynamics in Rat2 cells, measured by microtubule plus-end tracking behavior of CLIP-170-GFP after 1 h treatment with 0.1% DMSO or 100 nM nocodazole. Images in the left column show the first frame from the time-lapse movies. Images in the right column show maximum intensity projections from Z-stacks of all 600 frames. Scale bar = 10 µm. (**B**) Quantification of the number of all microtubule tracks per cell divided by cell area for CLIP-170-GFP. The data are expressed as the mean ± SEM. *N* = 3 independent experiments. *n* = 31 cells (DMSO), 25 cells (nocodazole). ***$p < 0.001$ (Mann-Whitney test). (**C**) Quantification of the mean track run length of CLIP-170-GFP comets per cell. The data are expressed as the mean ± SEM. *N* = 3 independent experiments. *n* = 31 cells (DMSO), 25 cells

(nocodazole). ***$p < 0.001$ (Mann-Whitney test). (**D**) Representative images from time-lapse movies of microtubule dynamics in Rat2 cells, measured by microtubule plus-end tracking behavior of EB3-GFP after 1 h treatment with 0.1% DMSO or 100 nM nocodazole. Images in the left column show the first frame from the time-lapse movies. Images in the right column show maximum intensity projections from Z-stacks of all 600 frames. Scale bar = 10 μm. (**E**) Quantification of the number of all microtubule tracks per cell divided by cell area for EB3-GFP. The data are expressed as the mean ± SEM. $N = 3$ independent experiments. $n = 23$ cells (DMSO), 24 cells (nocodazole). ***$p < 0.001$ (Mann-Whitney test). (**F**) Quantification of the mean track run lengths of EB3-GFP comets per cell. The data are expressed as the mean ± SEM. $N = 3$ independent experiments. $n = 23$ cells (DMSO), 24 cells (nocodazole). ***$p < 0.001$ (Mann-Whitney test).

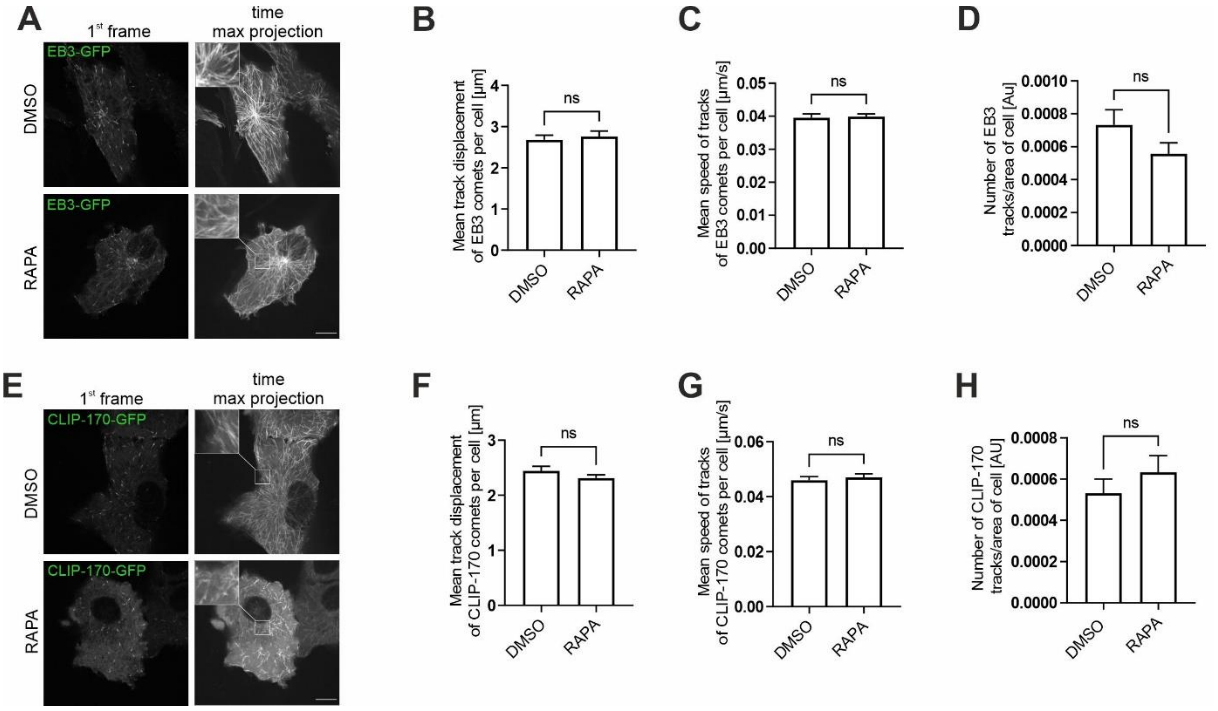

Fig. S7. Rapamycin does not affect microtubule dynamics in Rat2 cells. (**A**) Representative images from time-lapse movies of microtubule dynamics in Rat2 cells, measured by microtubule plus-end tracking behavior of EB3-GFP after 2 h treatment with 0.1% DMSO or 100 nM rapamycin (RAPA). Images in the left column show the first frame from the time-lapse

movies. Images in the right column show maximum intensity projections from Z-stacks of all 600 frames. Scale bar = 10 μm. (**B**) Quantification of the mean track run lengths of EB3-GFP comets per cell. The data are expressed as the mean ± SEM. *N* = 3 independent experiments. *n* = 27 cells (DMSO), 27 cells (RAPA). *ns*, nonsignificant (Mann-Whitney test). (**C**) Quantification of the mean speed of EB3-GFP comet. The data are expressed as the mean ± SEM. *N* = 3 independent experiments. *n* = 27 cells (DMSO), 27 cells (RAPA). *ns*, nonsignificant (Mann-Whitney test). (**D**) Quantification of the number of all microtubule tracks per cell divided by cell area for EB3-GFP. The data are expressed as the mean ± SEM. *N* = 3 independent experiments. *n* = 27 cells (DMSO), 27 cells (RAPA). *ns*, nonsignificant (Mann-Whitney test). (**E**) Representative images from time-lapse movies of microtubule dynamics in Rat2 cells, measured by microtubule plus-end tracking behavior of CLIP-170-GFP after 2 h treatment with 0.1% DMSO or 100 nM rapamycin (RAPA). Images in the left column show the first frame from the time-lapse movies. Images in the right column show maximum intensity projections from Z-stacks of all 600 frames. Scale bar = 10 μm. (**F**) Quantification of the mean track run lengths of CLIP-170-GFP comets per cell. The data are expressed as the mean ± SEM. *N* = 3 independent experiments. *n* = 35 cells (DMSO), 34 cells (RAPA). *ns*, nonsignificant (Mann-Whitney test). (**G**) Quantification of the mean speed of CLIP-170-GFP comet. The data are expressed as the mean ± SEM. *N* = 3 independent experiments. *n* = 35 cells (DMSO), 34 cells (RAPA). *ns*, nonsignificant (Mann-Whitney test). (**H**) Quantification of the number of all microtubule tracks per cell divided by cell area for CLIP-170-GFP. The data are expressed as the mean ± SEM. *N* = 3 independent experiments. *n* = 35 cells (DMSO), 34 cells (RAPA). *ns*, nonsignificant (Mann-Whitney test).

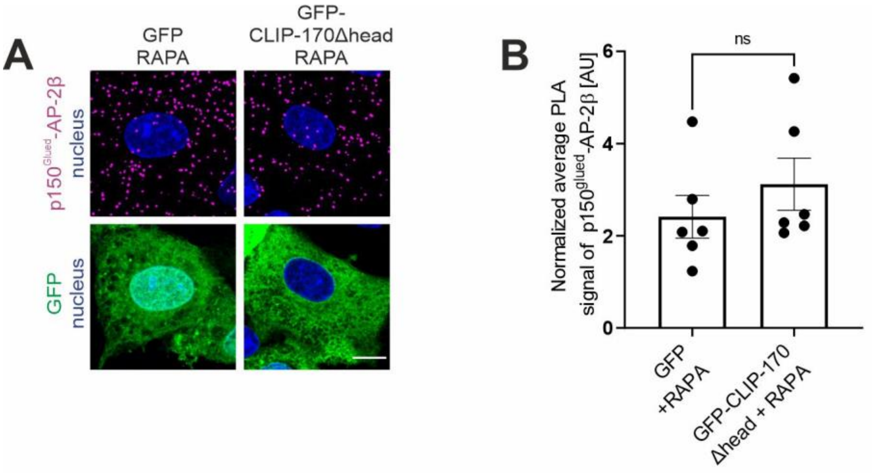

Fig. S8. Overexpression of dominant-negative mutant of CLIP-170 does not impact p150^{Glued}–AP-2β interaction. (**A**) Representative images of Rat2 fibroblasts that were transfected with pEGFPC1 or pEGFPC1-CLIP-170Δhead (green) and treated for 2 h with 100 nM rapamycin (RAPA) with PLA p150^{Glued}–AP-2β signals (magenta) and DAPI-stained nuclei (blue). Scale bar = 10 µm. (**B**) Quantification of the number of p150^{Glued}–AP-2β PLA puncta in cells that were treated as in A. The data are expressed as the mean number of PLA puncta per cell, normalized to the control variant (GFP + DMSO; not shown) ± SEM. *N* = 6 independent experiments. *n* = 94 cells (GFP + RAPA), 114 cells (GFP-CLIP-170Δhead + RAPA). *ns*, nonsignificant (Mann-Whitney test).

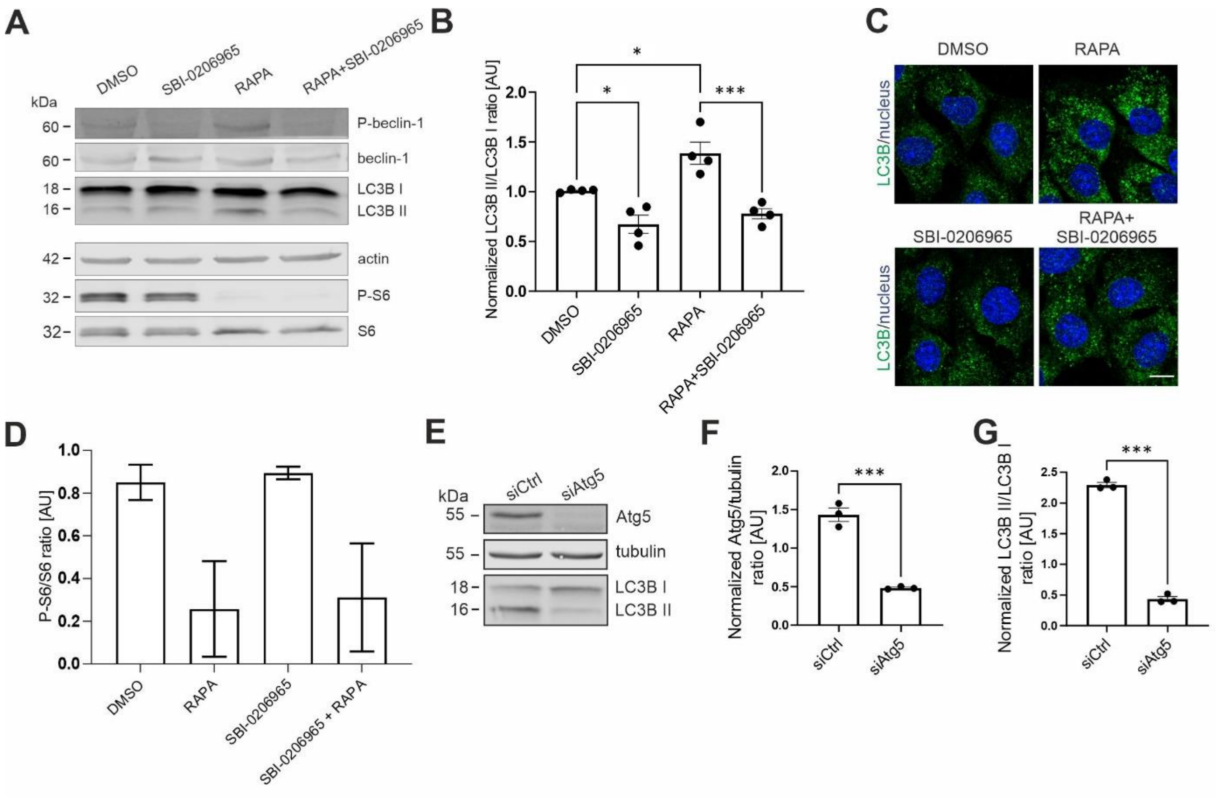

Fig. S9. Confirmation of effects of rapamycin, SBI-0206965, and Atg5 knockdown on autophagy. (**A**) Western blot analysis of endogenous P-beclin-1 (Ser30), beclin-1, LC3B I, LC3B II, actin, P-S6 (Ser235/236), and S6 levels in Rat2 fibroblasts that were treated with 0.1% DMSO for 2 h, 100 nM rapamycin (RAPA) for 2 h, 25 μM SBI-0206965 for 2 h 30 min, or 25 μM SBI-0206965 for 30 min and 100 nM rapamycin for 2 h (RAPA + SBI-0206965). (**B**) Densitometry analysis of normalized LC3B II/LCB I ratio in Rat2 cells that were treated as in A. The data are presented as mean of the normalized ratio of LC3B II to LC3B I levels ± SEM. $N = 4$ independent experiments. *$p < 0.05$, ***$p < 0.001$ (one-way ANOVA followed by Bonferroni multiple-comparison *post hoc* test). (**C**) Representative images of Rat2 cells that were treated with 0.1% DMSO for 2 h, 100 nM rapamycin (RAPA) for 2 h, 25 μM SBI-0206965 for 2 h 30 min, or 25 μM SBI-0206965 for 30 min and 100 nM rapamycin for 2 h (RAPA + SBI-0206965), with immunofluorescently labeled endogenous LC3B (green) and nuclei that were stained with Hoechst 33258 (blue). Scale bar = 10 μm. (**D**) Quantitative analysis of endogenous P-S6 (Ser235/236)/S6 levels ratio (analyzed by Western blot, see Fig. 4A) in

HEK293T cells that were treated with 0.1% DMSO for 2 h, 100 nM rapamycin (RAPA) for 2 h, 25 μM SBI-0206965 for 2 h 30 min, or 25 μM SBI-0206965 for 30 min and 100 nM rapamycin for 2 h (RAPA + SBI-0206965). *N* = 2 independent experiments. (B) (**E**) Western blot analysis of endogenous Atg5, LC3B I, LC3B II, and tubulin in Rat2 cells that were transfected with siCtrl or rat siAtg5 for 72 h. (**F**) Densitometry analysis of normalized Atg5 in Rat2 cells that were treated as in D. The data are presented as mean of the normalized ratio of Atg5 to tubulin levels ± SEM. *N* = 3 independent experiments. ***$p < 0.001$ (Student's *t*-test). (**G**) Densitometry analysis of normalized LC3B II/LCB I ratio in Rat2 cells that were treated as in D. The data are presented as mean of the normalized ratio of LC3B II to LC3B I levels ± SEM. *N* = 3 independent experiments. ***$p < 0.001$ (Student's *t*-test).

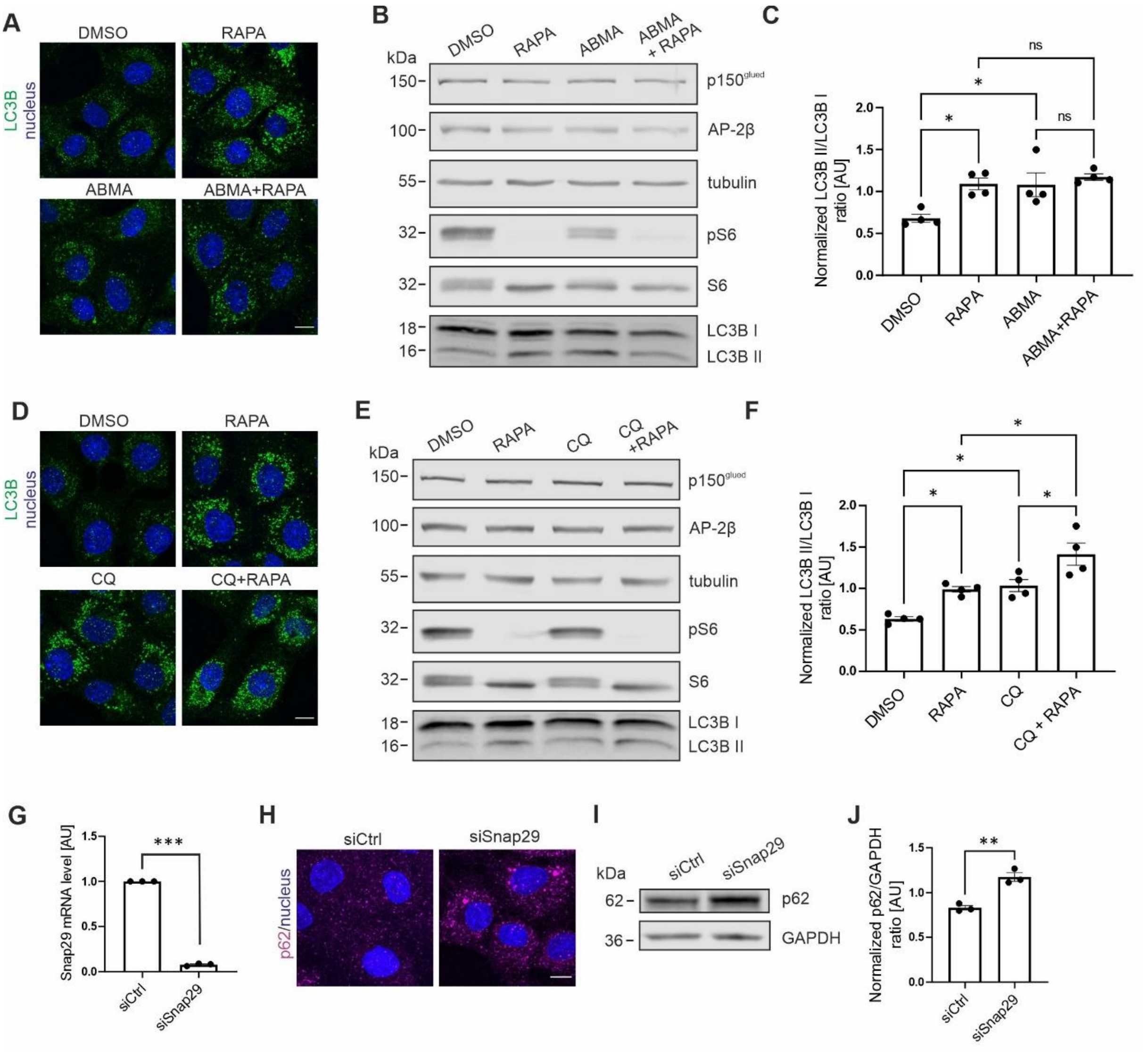

Fig. S10. Confirmation of effects of ABMA, CQ, and SNAP29 knockdown on autolysosomal pathway. (**A**) Representative images of Rat2 cells that were treated for 2 h with 0.1% DMSO, 100 nM rapamycin (RAPA), 60 μM ABMA (ABMA), or 60 μM ABMA and 100 nM rapamycin (ABMA+RAPA), with immunofluorescently labeled endogenous LC3B (green) and nuclei that were stained with Hoechst 33258 (blue). Scale bar = 10 μm. (**B**) Western blot analysis of endogenous proteins levels as indicated in Rat2 cells that were treated as in A. (**C**) Densitometry analysis of normalized LC3B II/LCB I ratio in Rat2 cells that were treated as in A. The data are presented as mean of the normalized ratio of LC3B II to LC3B I levels ± SEM. $N = 4$ independent experiments. $*p < 0.05$, *ns* – nonsignificant (one-way ANOVA followed by Bonferroni *post hoc* test). (**D**) Representative images of Rat2 cells that were treated for 2 h with 0.1% DMSO, 100 nM rapamycin (RAPA), 50 μM chloroquine (CQ), or 50 μM chloroquine and 100 nM rapamycin (CQ+RAPA), with immunofluorescently labeled endogenous LC3B (green) and nuclei that were stained with Hoechst 33258 (blue). Scale bar = 10 μm. (**E**) Western blot analysis of endogenous protein levels as indicated in Rat2 cells that were treated as in D. (**F**) Densitometry analysis of normalized LC3B II/LCB I ratio in Rat2 cells that were treated as in D. The data are presented as mean of the normalized ratio of LC3B II to LC3B I levels ± SEM. $N = 4$ independent experiments. $*p < 0.05$ (one-way ANOVA followed by Bonferroni *post hoc* test). (**G**) Results of qRT-PCR analysis of Snap29 mRNA levels relative to tubulin mRNA in Rat2 cells that were transfected with control siRNA (siCtrl) or siRNA against Snap29 (siSnap29) for 72 h. The results are expressed as means, normalized to control ± SEM. $N = 3$ independent experiments. $***p < 0.001$ (one-sample *t*-test). (**H**) Representative images of Rat2 cells that were treated as in G, with immunofluorescently labeled endogenous p62/SQSTM1 (magenta) and nuclei that were stained with Hoechst 33258 (blue). Scale bar = 10 μm. (**I**) Western blot analysis of endogenous protein levels as indicated in Rat2 cells that were treated as in G. (**J**) Densitometry analysis of normalized p62/SQSTM1 in Rat2 cells that were treated

as in G. The data are presented as mean of the normalized ratio of p62 to tubulin levels ± SEM. $N = 3$ independent experiments. $^{**}p < 0.01$ (Student's t-test).

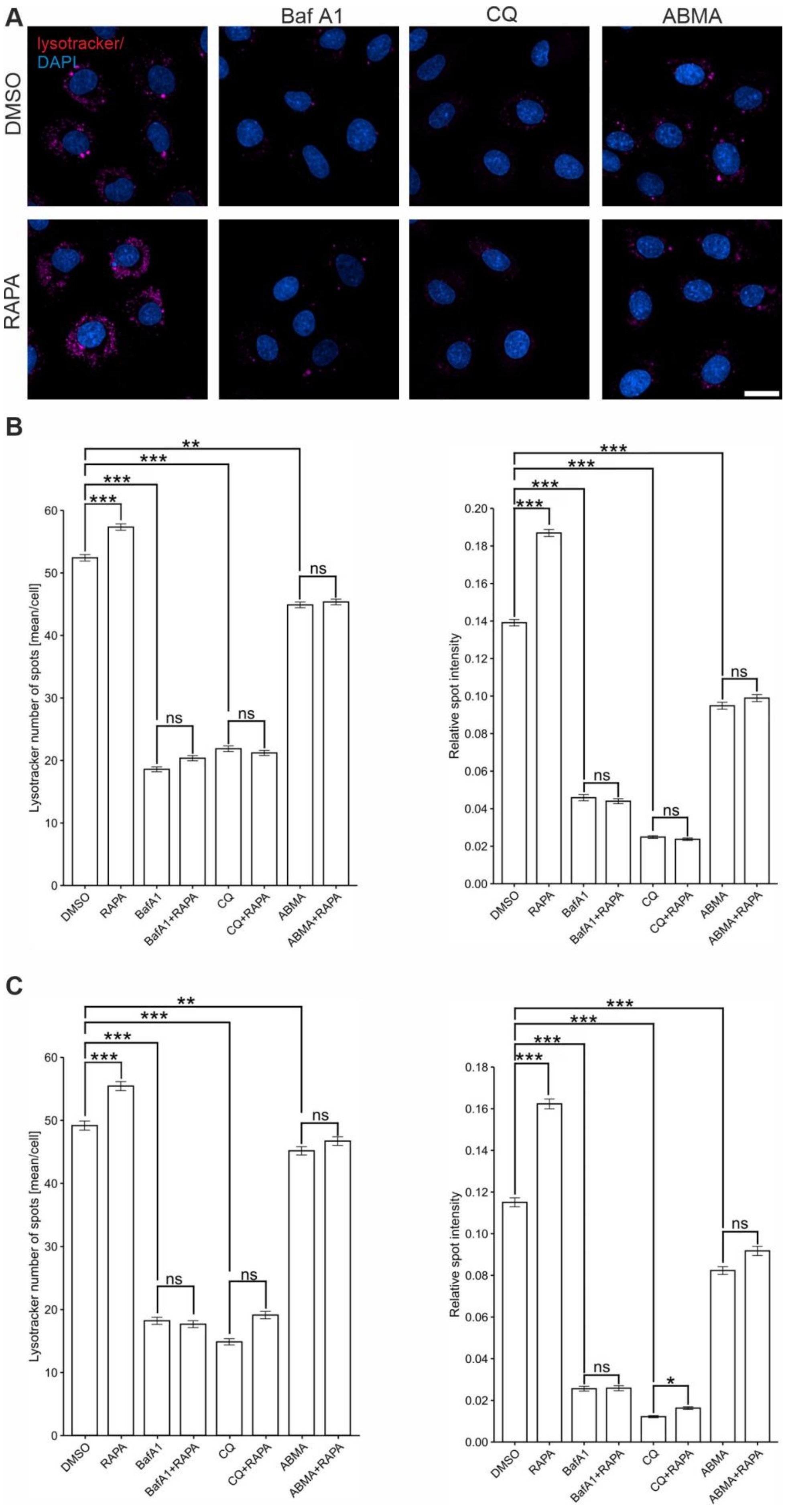
A
Baf A1
CQ
ABMA
lysotracker/
DAPI
DMSO
RAPA
B
Lysotracker number of spots [mean/cell]
Relative spot intensity
DMSO
RAPA
BafA1
BafA1+RAPA
CQ
CQ+RAPA
ABMA
ABMA+RAPA
ns
***
**
C
Lysotracker number of spots [mean/cell]
Relative spot intensity
*

Fig. S11. (**A**) Representative images of Rat2 cells that were treated for 2 h as indicated, with immunofluorescently labeled Lysotracker (magenta) and nuclei that were stained with Hoechst 33258 (blue). Scale bar = 20 μm. (**B, C**) Quantification of Lysotracker mean number of spots per cell and relative spot intensity in cells that were treated with 0.1% DMSO, 100 nM rapamycin (RAPA), 100 nM bafilomycin A1 (BafA1), 50 μM chloroquine (CQ), 60 μM ABMA (ABMA), or a combination of 100 nM rapamycin with 100 nM bafilomycin A1 (BafA1 + RAPA), 50 μM chloroquine (CQ + RAPA), or 60 μM ABMA (ABMA + RAPA). The results are expressed as the mean ± SEM. $N = 2$ independent experiments. $n = 913$ and 689 cells analyzed in each group for repetition 1 (passage 4, B) and 2 (passage 7, C). *$p < 0.05$, **$p < 0.01$, **$p < 0.001$, *ns* – nonsignificant (Kruskal-Wallis test followed by Dunn's *post hoc* test). Results from both independent biological repetitions of high-throughput microscopy analyses are presented.

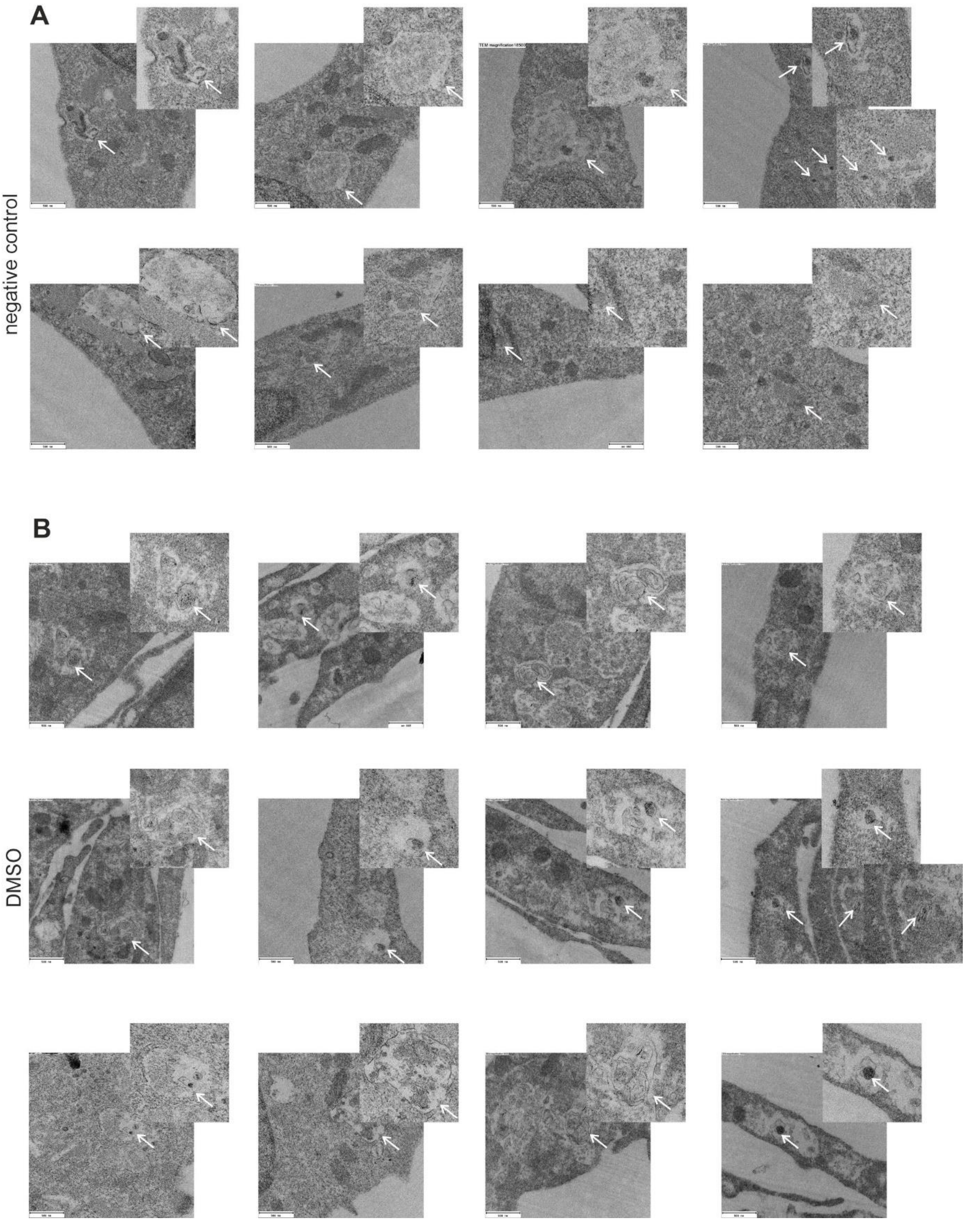
A
negative control
B
DMSO

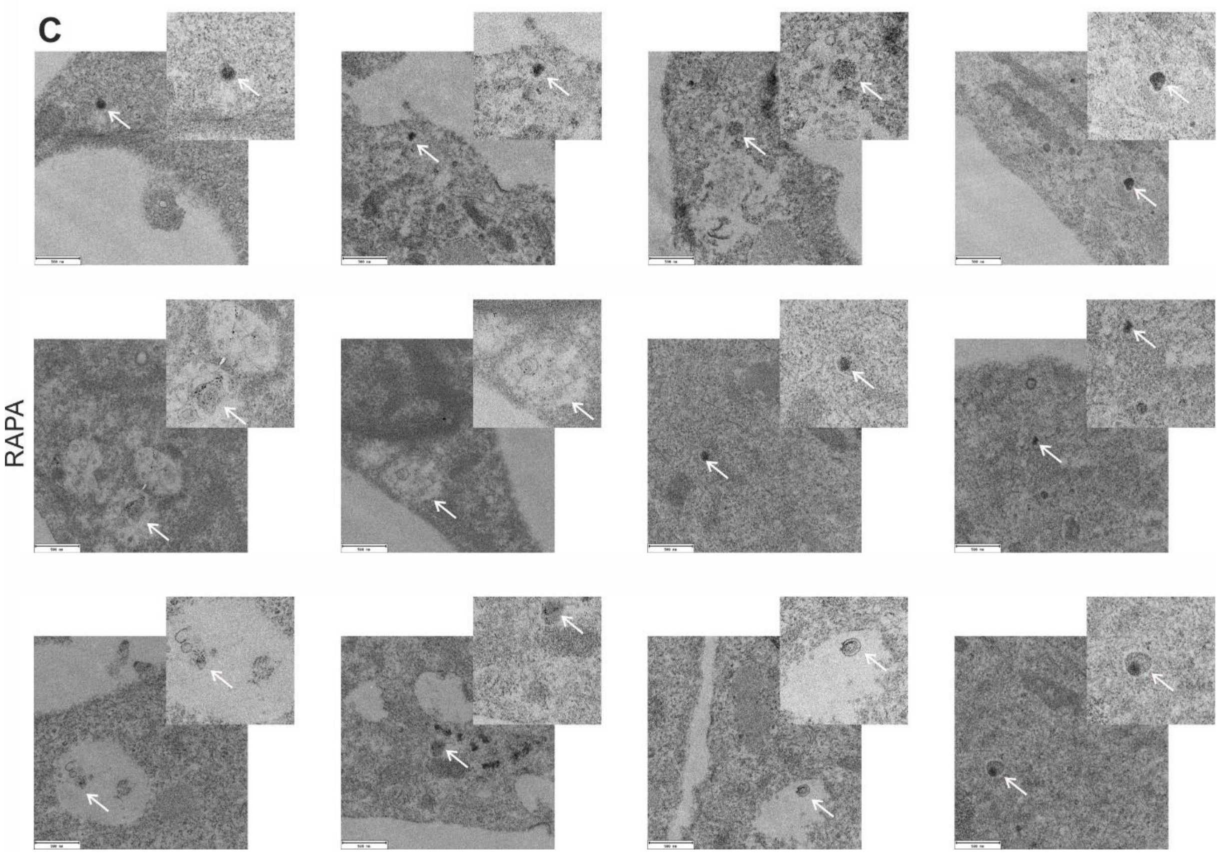

Fig. S12. Additional representative p150^{Glued}–AP-2β PLA-EM images of Rat2 fibroblasts (**A**) negative control without primary antibodies. (**B**) cells treated with 0.1% DMSO for 2 h (**C**) cells treated with 100 nM rapamycin (RAPA) for 2 h. White arrows point to PLA signals that co-occurred with organelles that resembled lysosomes or autophagosomes. Black boxes indicate the regions shown in higher magnification. Scale bar = 500 nm.

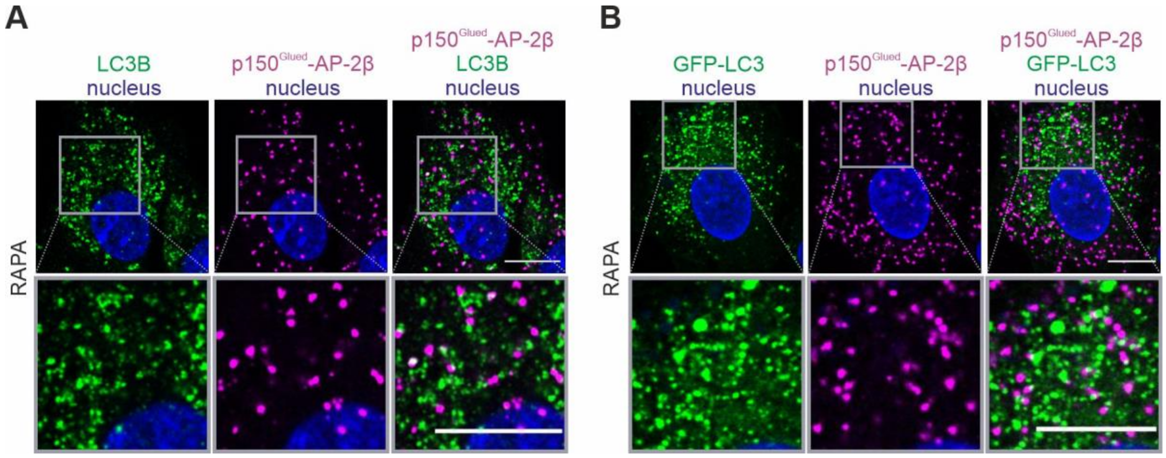

Fig. S13. $p150^{Glued}$–AP-2β PLA signal co-localizes poorly with LC3. (**A**) Representative images of Rat2 cells treated with 100 nM rapamycin (RAPA) with PLA $p150^{Glued}$–AP-2β signals (magenta) immunofluorescently labeled for endogenous LC3B (green) and nuclei stained with DAPI (blue). Images were acquired using the AiryScan module. Scale = 10 μm. (Upper panel) Representative photograph of a single cell. (Lower panel) Close-up of a site with PLA and LC3B signals. (**B**) Representative images of Rat2 cells transfected with GFP-LC3 for 24 h and then treated with 100 nM rapamycin (RAPA) with PLA $p150^{Glued}$–AP-2β signals (magenta), overproduced GFP-LC3 (green), and nuclei stained with DAPI (blue) and. Images were acquired using the AiryScan module. Scale = 10 μm. (Top) Representative photograph of a single cell. (Bottom) Close-up of a site with PLA and GFP-LC3B signals.

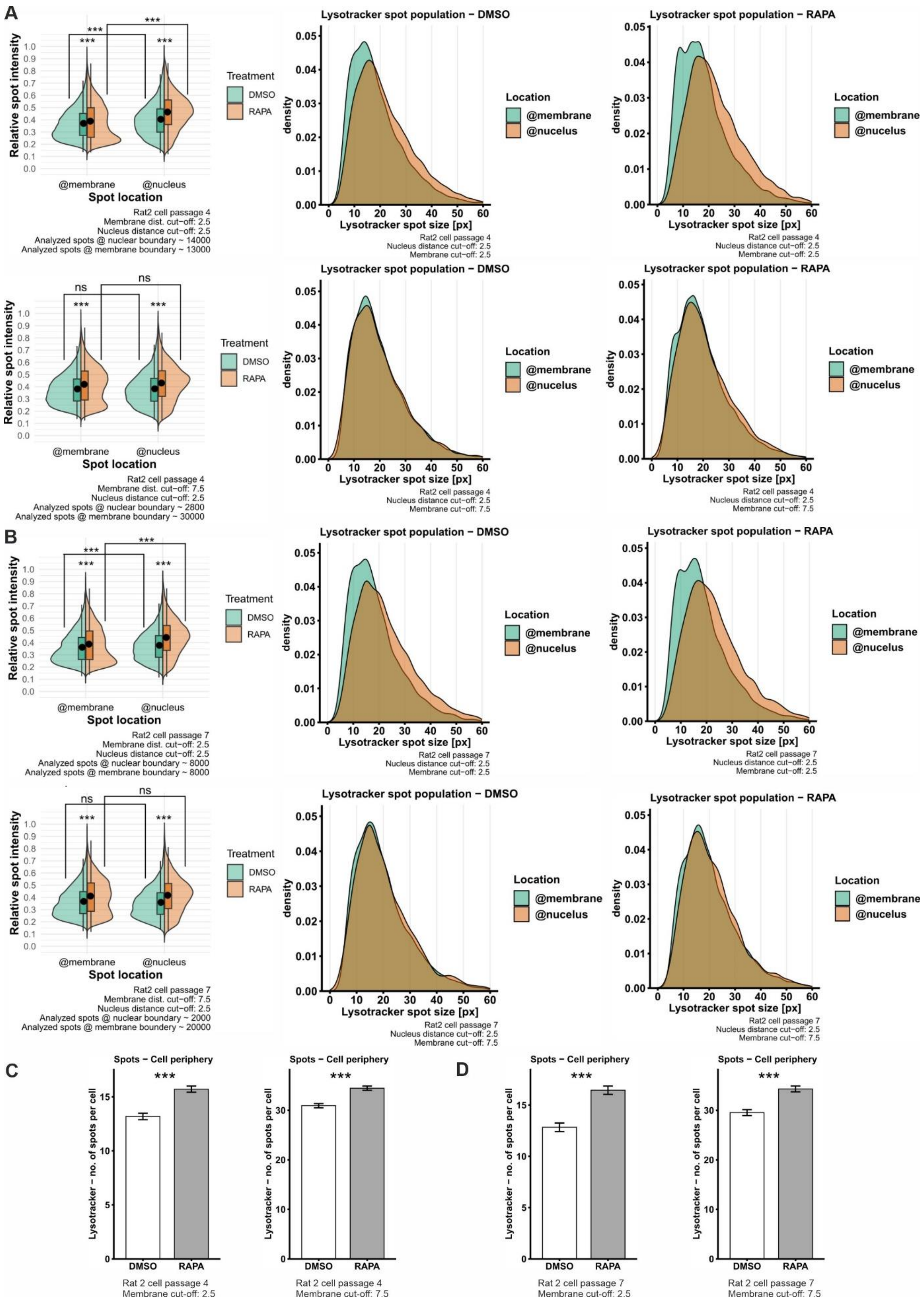

Fig. S14. Results of analysis of lysotracker staining distribution and intensity in cells treated with 0.1% DMSO or 100 nM rapamycin for 2 h. The images from the experiments

described in Fig. S11 were re-evaluated for fluorescence intensity and number of lysotracker objects in relation to cellular distribution. The peripheral pool was defined as objects within 2.5 μm or 7.5 μm of the plasma membrane. The perinuclear pool was defined as objects within 2.5 μm of the nucleus. (**A**) Results of lysotracker peripheral pool fluorescence intensity analysis in Repetition 1 of the experiment (passage 4). (**B**) Results of lysotracker peripheral pool fluorescence intensity analysis in Repetition 2 of the experiment (passage 7). (**C**) Results of lysotracker peripheral pool objects number analysis for Repetition 1 of the experiment (passage 4). (**D**) Results of lysotracker peripheral pool objects number analysis in Repetition 2 of the experiment (passage 7). ***$p < 0.001$ (Student's *t*-test).

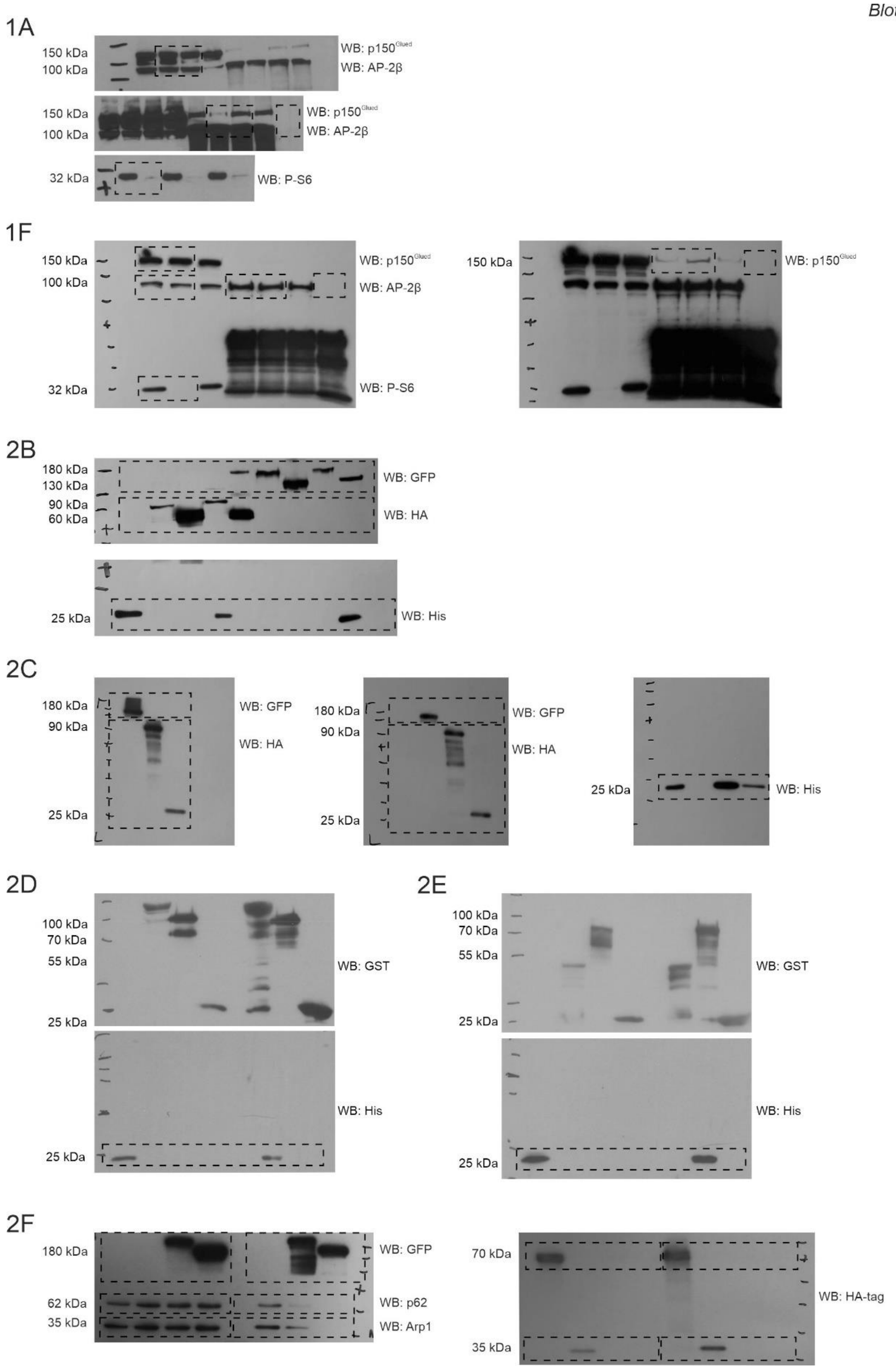
1A
150 kDa
100 kDa
WB: p150Glued
WB: AP-2β
150 kDa
100 kDa
WB: p150Glued
WB: AP-2β
32 kDa
WB: P-S6
1F
150 kDa
100 kDa
32 kDa
WB: p150Glued
WB: AP-2β
WB: P-S6
150 kDa
WB: p150Glued
2B
180 kDa
130 kDa
90 kDa
60 kDa
WB: GFP
WB: HA
25 kDa
WB: His
2C
180 kDa
90 kDa
25 kDa
WB: GFP
WB: HA
180 kDa
90 kDa
25 kDa
WB: GFP
WB: HA
25 kDa
WB: His
2D
100 kDa
70 kDa
55 kDa
25 kDa
WB: GST
WB: His
25 kDa
2E
100 kDa
70 kDa
55 kDa
25 kDa
WB: GST
WB: His
25 kDa
2F
180 kDa
62 kDa
35 kDa
WB: GFP
WB: p62
WB: Arp1
70 kDa
35 kDa
WB: HA-tag

2G

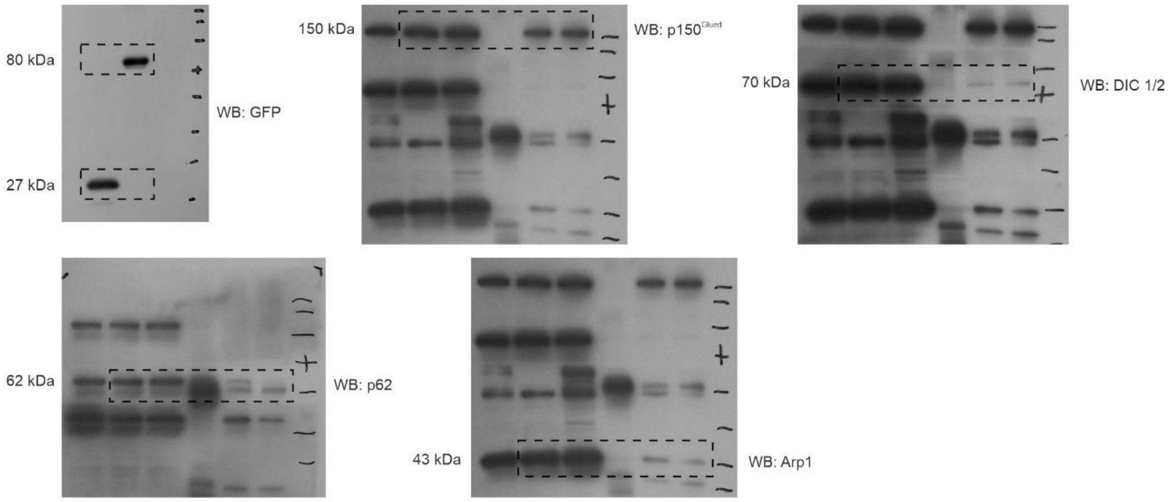
80 kDa
27 kDa
WB: GFP
150 kDa
WB: p150Glued
70 kDa
WB: DIC 1/2
62 kDa
WB: p62
43 kDa
WB: Arp1

3A

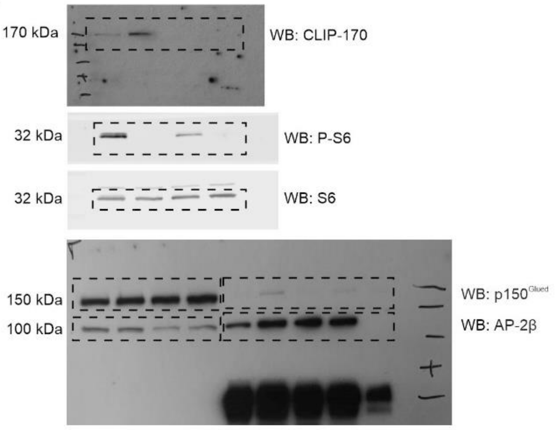
170 kDa
WB: CLIP-170
32 kDa
WB: P-S6
32 kDa
WB: S6
150 kDa
100 kDa
WB: p150Glued
WB: AP-2β

3B

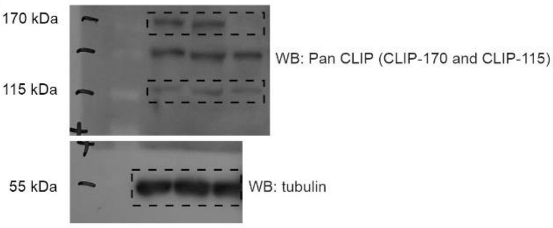
170 kDa
115 kDa
WB: Pan CLIP (CLIP-170 and CLIP-115)
55 kDa
WB: tubulin

4A

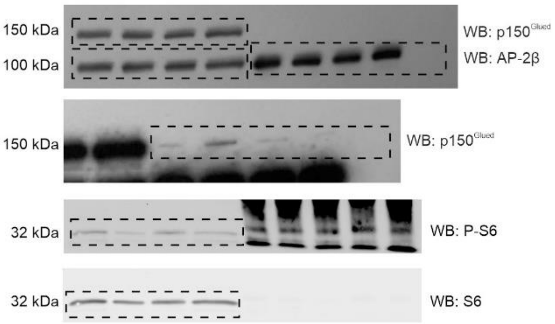
150 kDa
100 kDa
WB: p150Glued
WB: AP-2β
150 kDa
WB: p150Glued
32 kDa
WB: P-S6
32 kDa
WB: S6

5A

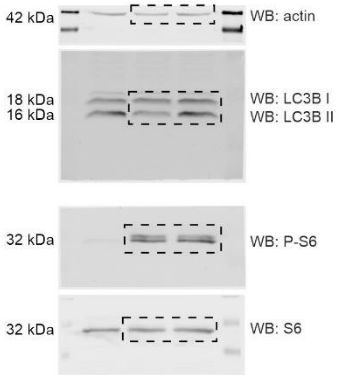
42 kDa
WB: actin
18 kDa
16 kDa
WB: LC3B I
WB: LC3B II
32 kDa
WB: P-S6
32 kDa
WB: S6

5F

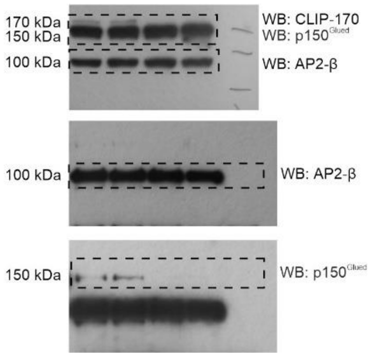

5I

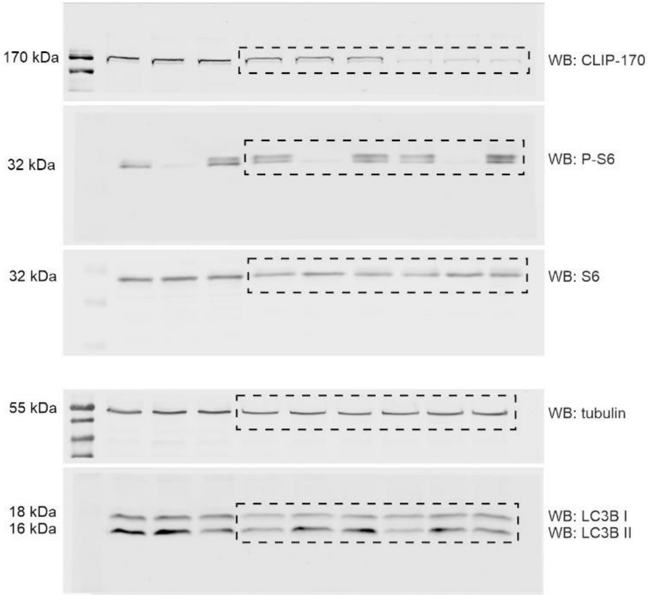

S4A

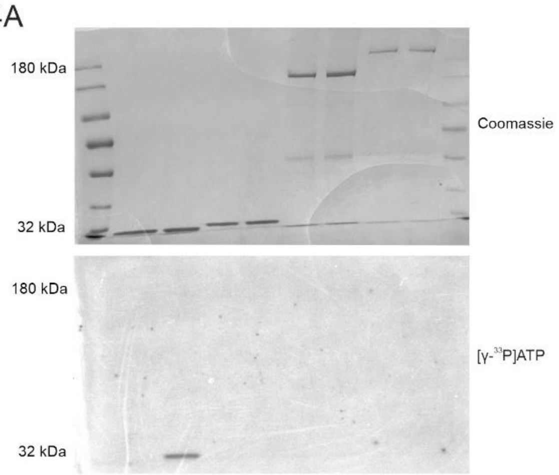

S4B

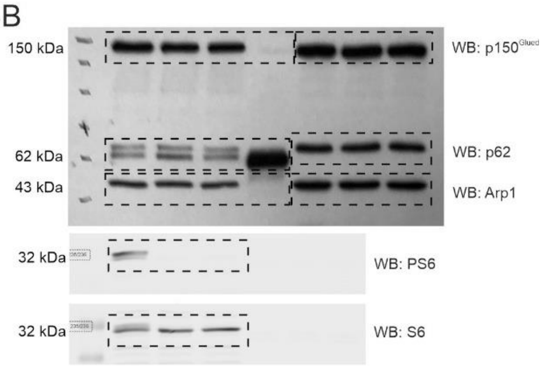

S9A

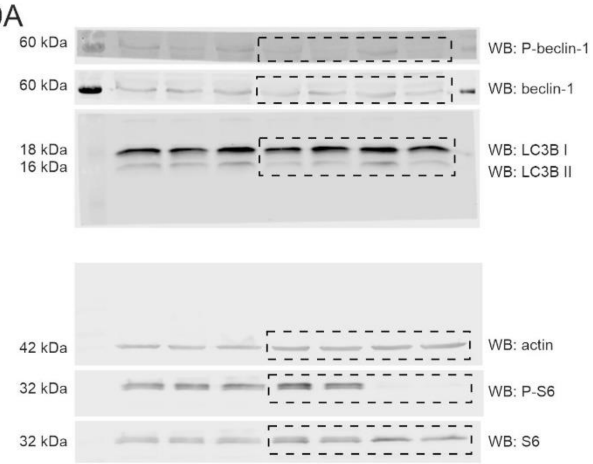

S9D

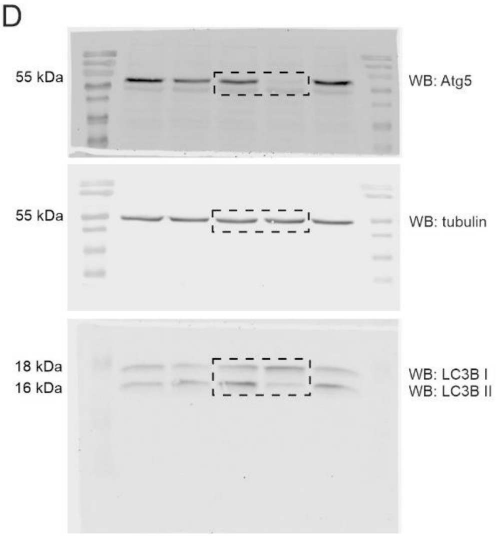

S10B

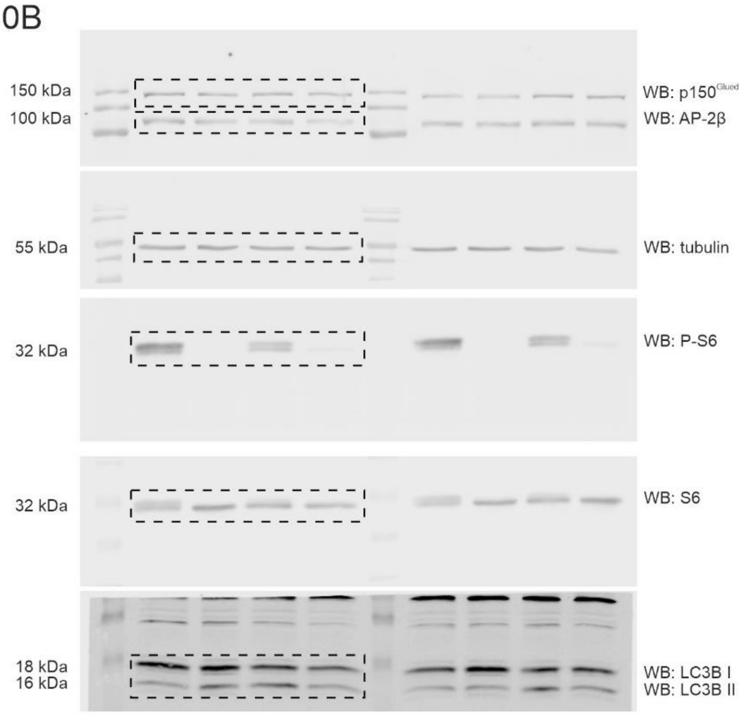

S10E

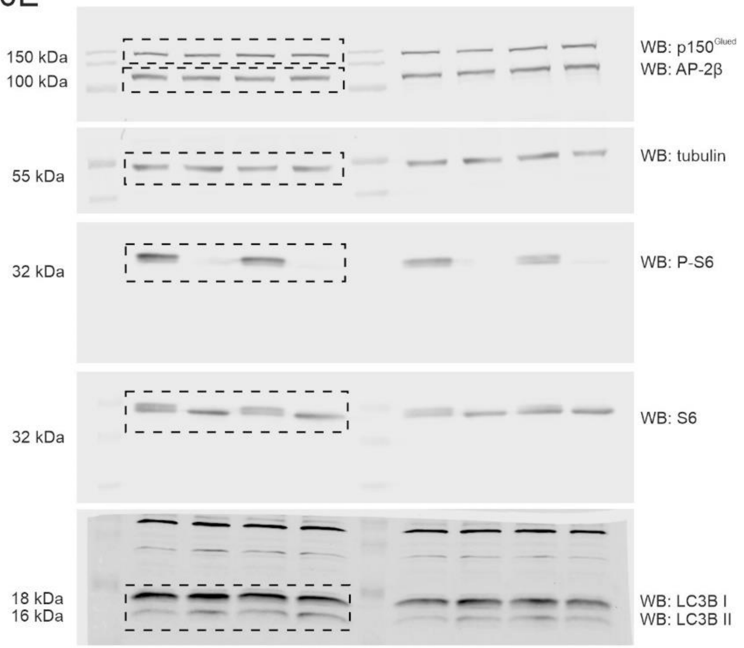

S10I

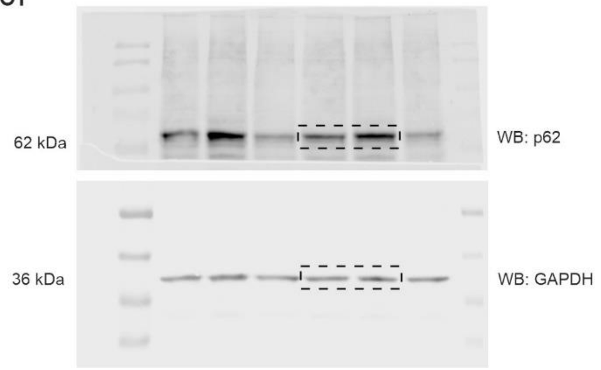

Fig. S15. Blot transparency. Full-size blots that correspond to cropped images that are presented in the manuscript.
